# The existential stakes of platform governance and online content regulation: a critical conceptual model

**DOI:** 10.12688/openreseurope.13358.1

**Published:** 2021-03-31

**Authors:** Charilaos Papaevangelou

**Affiliations:** 1 JOLT-ETN / LERASS, University Paul Sabatier - Toulouse III, Toulouse, France

**Keywords:** Platform governance, online content regulation, multi-stakeholder governance, social media regulation, regulatory governance

## Abstract

This study introduces a comprehensive yet non-exhaustive overview of literature concerning the concepts of regulation and governance, and attempts to connect them to scholarly works that deal with social media platforms’ content regulation. The paper provides fundamental definitions of regulation and governance, along with a critique of polycentricity, in order to contextualise the discussion around platform governance and online content regulation. Regulation is framed here as a governance mechanism within a polycentric governance model where stakeholders have competing interests, even if sometimes they coincide. Moreover, where traditional governance literature conceptualised stakeholders as a triangle, this article proposes imagining them as overlapping circles of governance clusters with competing interests, going beyond the triad of public, private and non-governmental actors. Finally, the paper contends that that there exists a timely need to reimagine the way in which we understand and study phenomena appertaining to public discourse by adopting the platform governance perspective, which is framed as the advancement of internet governance. Finally, the article ascertains to study the governance of online content and social media platforms not as a sub-section of internet governance but as a conceptual evolution with existential stakes.

## Plain language summary

This article examines academic literature regarding the notions of regulation and governance, trying to define what they mean, how they are used depending on the field of application and how they are framed specifically when studying online platforms (e.g., Facebook, Google, etc.). The author begins by acknowledging that, while this is not an exhaustive research, there has been a wide embrace of terms like “platform governance” and “online content regulation,” even by policymakers. Therefore, the author is interested in defining what these concepts mean and how they can be used to study online platforms. The author also provides a brief historical retrospective on how academics have studied the way that the internet is structured and governed by participating stakeholders. Finally, the author concludes that, whereas in the early 1990s cyber-utopians imagined the internet to be a democratised, decentralised and self-regulated space, away from state interventions, we are now in the age of platform governance. Platform governance is a term inherently connected with the multiplicity and plurality of stakeholders but places online platforms at the epicentre. This is quite useful because it allows us to better engage with platforms and, specifically, social media and infomediaries. So, where internet governance began by celebrating independence, platform governance begins by celebrating collaborations with a myriad of stakeholders, including states. Finally, the paper argues that by being able to discern these notions, we are better equipped to reimagine how regulatory frameworks should be designed, especially those that are related to online content, which constitutes a large part of our online discussions on social media and elsewhere.

## Introduction

Recently, a significant volume of scholarly work has embraced the burgeoning use of notions like *platform governance* ( Caplan & Gillespie, 2020; Fan & Zhang, 2020; Gorwa, 2019b; Haggart, 2020; Mazzucato *et al*., 2020; Napoli, 2015) and *online content regulation* ( Douek, 2021; Gorwa, 2019a; Land, 2019). This has further expanded the interdisciplinary boundaries of literature that relate to regulation and governance. The underlying common ground of these works is online platforms and, specifically, social media or infomediation platforms ( Bucher & Helmond, 2018; Gillespie, 2010; Smyrnaios & Rebillard, 2019). However, there has yet to be a reflective analysis of these newly employed terms and, specifically, an attempt to connect them to critical theories, such as the one proposed by Madeline Carr as regards multi-stakeholder governance ( Carr, 2015).

To this end, this paper seeks to theoretically frame the discussion with works coming from the broader field of regulation and governance ( Braithwaite *et al*., 2007; Kjaer & Vetterlein, 2018; Levi-Faur, 2011), internet governance ( DeNardis, 2020; Hofmann *et al*., 2016), and, ultimately, to connect it to research studying content moderation or regulation of social media platforms ( Douek, 2019; Flew *et al*., 2019; Gorwa, 2019a). Therefore, this paper acts as a critical exploration of relevant literature, aspiring primarily to help online media scholars to navigate the multifaceted domain of regulation. The paper is structured in the following way: the first section defines regulation and governance, the second section defines polycentric or multi-stakeholder governance regimes, and the final part discusses the trending notions of platform governance and online content regulation.

The works considered here are meant to be representative of relevant literature and their selection was done in an organic way (i.e., they were selected through a conceptual connection by following papers’ citations and theoretical concepts). This article is by no means an exhaustive piece of research, but rather it is an invitation to investigate the interdisciplinarity and depths of an emerging yet vibrant field, which seeks to understand the governance of online platforms and their regulation, as well as their implications relating to democracy and public discourse. This is a truly important field not only because it expands our research horizons, but also because it aims to inform stakeholders found at every position within the governance spectrum. It is thus a timely effort to properly situate the discussion revolving around online content regulation to be better equipped to tackle it.

## Towards a definition of regulation and governance

### Regulation

Attempting to define something as elusive and broad as regulation can be a complicated task. Regulation consists of a large gamut of factors, including “politics, policies, institutions and effectiveness of formal and informal controls” ( Levi-Faur, 2011, p. 16). Therefore, in order to study regulation, one has to take into consideration a plethora of elements, alongside their innate political and, often, conflictual attributes. David-Levi Faur offers us a comprehensive overview of the nascent multidisciplinary field of regulation in his seminal book *Handbook on the Politics of Regulation* (2011), inviting us to consider how regulation can be framed depending on the field of research. Regulation, in recent years, has become a distinct field of international practice and research, especially after the introduction of the economic theory of regulation ( Stigler, 1971). Certainly, the definition of regulation varies even among economic theorists: some argue that it acts as another weapon of neoliberalism against the welfare state ( Majone, 1994), while others believe it to be an important tool to fuel competition ( Levi-Faur, 2011, p. 3).

In any case, the concept of regulation expands well beyond the theory of economy and covers the field of standard-setting and administration. Some scholars have talked about the benefits of regulation against consumer exploitation, environmental misdoings and other activities in a rather pragmatistic way ( Koop & Lodge, 2015; Marx, 2011). Moreover, one could not neglect adding to this long interdisciplinary list, the framing of regulation by social and political sciences as a means of control ( Beresford, 2003; Levi-Faur, 2011, pp. 3, 16) that, inter alia, seeks to dictate a change in behaviour ( Koop & Lodge, 2015). It is, thus, clear that there is not one single definition for regulation. Levi-Faur frames it as “the *ex-ante* bureaucratic legalisation of prescriptive rules and the monitoring and enforcement of these rules by social, business, and political actors on other social, business, and political actors” ( Levi-Faur, 2011, p. 6; emphasis theirs).

Consequently, this is a definition with a distinct administrative approach to regulation, while excluding the “legislative or judicial rule making” (ibid). Elsewhere, Koop and Lodge frame regulation as following: "[it is the] intentional intervention in the activities of a target population, where the intervention is typically direct – involving binding standard-setting, monitoring, and sanctioning – and exercised by public-sector actors on the [activities] of private-sector actors" ( Koop & Lodge, 2015, p. 106). The two definitions share the same characteristics concerning how regulation works (i.e., standard-setting and not rule-making, monitoring and enforcement). However, while the former definition highlights the *consequentiality* of regulation, the latter emphasises the significant notion of *intentionality*, which echoes the *interventionist* tradition of regulation ( Levi-Faur, 2011, p. 4).

Nevertheless, the fundamental common point of the definitions is the development of targeted and binding rules, which Black purports aim to “change the behaviour of others […] through a combination of rules and norms” ( Black, 2008, p. 139). As a result, we can further distinguish regulation according to its implications, resonating once more with the consequentialist approach. So, on the one hand, there is regulation that serves the “public interest” ( Hofmann *et al*., 2016, p. 1410; Levi-Faur, 2011, p. 28) and, on the other, regulation that “mainly serves private interests,” which some have called “deregulation” ( Levi-Faur, 2011, p. 28). It is made, thus, visible that the envisioned goal of regulation as beneficial to the public interest is by no means a given; it is hard to argue that all actors in a competing environment share the same values. It should be also noted that a “public-interest” approach has gained significant traction within the field of media studies ( Napoli, 2015; van Dijck *et al*., 2018), as well as within governance literature following a human-rights based approach ( Kaye, 2019; Land, 2019).

This is why it is very important to acknowledge that regulation is itself a product of negotiations and power dynamics. Therefore, while regulation concerns primarily ex-ante standard-setting or rules, following a consequentialist approach, it is impossible to predict its outcome, but merely gauge its impact. To this end, some argue that the key way of mitigating such regulatory risk is the multi-stakeholder governance model ( Black, 2008). In other words, regulation that is developed by a single authority with specific results in mind is less flexible and, thus, less effective when dealing with ever-everchanging phenomena; hence, polycentricity is often framed as panacea, which has come to monopolise the way of analytically framing the discussion revolving around governance ( Carr, 2015; Hofmann, 2020). At any rate, as relevant literature attests, recent governance regimes include a multitude of different stakeholders deliberating regulatory frameworks, which has accelerated the decentralisation of state power ( Abbott & Snidal, 2009; Bernstein & Cashore, 2007; Büthe & Mattli, 2011; Levi-Faur, 2011; Majone, 1994).

Furthermore, the actors most commonly met within these power structures are: state actors, non-state or market actors, and non-governmental or civil actors ( Abbott & Snidal, 2009, pp. 8–10; Gorwa, 2019a, p. 2; Levi-Faur, 2011, p. 10). Accordingly, three types of regulation are most commonly met in the relevant literature: self-regulation, co-regulation, and top-down (or ‘command-and-control’) regulation ( Gorwa, 2019b, p. 853; Hirsch, 2013; Levi-Faur, 2011, p. 531; Marsden, 2011, pp. 13–14):

• **Self-regulation**: This type of regulation refers primarily to non-state, “voluntary and ‘non-binding’” agreements and principles ( Gorwa, 2019b), such as platforms’ “Terms of Services” ( Bietti, 2020; Suzor, 2019) or self-organised industry groups, such as the “Global Internet Forum to Counter Terrorism” ( Gorwa, 2019b). This type of regulation is by and large preferred by firms as it greatly reduces costs of implementing formal legislation, which has also given way to the privatization of regulation ( Büthe & Mattli, 2011). Moreover, this type of regulation has little legitimacy in polycentric regimes, as it is tied to a *laissez-faire* attitude ( Bernstein & Cashore, 2007; Büthe & Mattli, 2011; Flew *et al*., 2019; Marsden, 2011), which often lacks legal repercussions. Moreover, self-regulation seeks to consolidate an actor’s (or a cluster of actors) self-governance, that is, their independence of a hierarchically higher authority to hold them to account.

• **Co-regulation**: This type of regulation primarily refers to the attempt of combining the ‘best’ of all three actors’ competencies, which Abbott and Snidal argue are: “interdependence, representativeness, expertise, and operational capacity” (p. 66). We could argue that this type of regulation acts as the cornerstone of the polycentric regime and is thus often depicted as essential to democratic representation and plurality ( Black, 2008; Cammaerts & Mansell, 2019). However, each actor has its own agenda, making contention unavoidable. A large number of scholars, policymakers and, recently, online platforms, are in favour of this type of regulation, also called as “soft-law” ( Mattli & Woods, 2009, p. 1), because it “[opens up a] more interesting [conversation] than a static no-regulation versus state regulation binary choice” ( Marsden, 2011, p. 242). Co-regulation seeks to consolidate a shared governance (co-governance) among stakeholders. Accountability here varies but, in most cases, it takes the shape of periodic transparency reports, audits, and repercussions in cases where notice isn’t followed by action.

• **Top-down regulation**: Last, self-regulation refers to state regulation, which is usually passed by public authorities in the form of official legislation, or “hard rules” ( Mattli & Woods, 2009, p. 1), often directly intervening in an industry or a market. State regulation is usually critiqued as cumbersome and counterproductive, especially concerning innovation ( Bostoen, 2018). However, it can work as the “baseline” ( Gorwa, 2019b, p. 8) upon which other types of regulation are built, “either as complements to fill in certain gaps, or as substitutes to proposals perceived as overly invasive or harmful to human rights” (ibid). Its legitimacy can vary depending on the state which regulates and the political state of affairs (e.g., democratic processes, political representation, etc.). Accountability is high because there are legal consequences to actors who do not abide by the state’s regulation and it is the state that will hold to account a rogue actor. However, it should be noted that this too is to be taken with a grain of salt because, on the one hand, the state has its own agenda (e.g., to satisfy electorates), and, on the other, because the state itself might avoid accountability due to authoritative concentration of power.

Levi-Faur adds some nuances to the traditional typology: according to him, “pure self-regulation” (p. 531) is a branch of “[hybrid] meta-regulation,” which refers to a confined role of the regulator to the “institutionalisation and monitoring” of standards and rules (p. 11). He also adds another type of regulation, that of “[hybrid] multi-level regulation,” emphasising the geopolitical implications of regulators, where the “regulatory authority is allocated to different levels of territorial tiers” (ibid). We believe that while the latter may add an important nuance to critical analyses, the former rather complexifies the discussion; conversely, we propose restricting meta-regulation to that, which “regulates any other form of regulation” (Parker in Levi-Faur, 2011, p. 11). In any case, one can easily discern when going through the typology of regulations that the overarching notion is inherently connected to the concept of governance.

As a result, many scholars have been increasingly treating regulation and governance almost synonymously ( Hofmann *et al*., 2016). First, we ought to unpack these two notions and be mindful of their distinctions. We should underline that regulation and governance are not synonymous; treating them as such “[strips regulation] of some analytical potential” (p. 6) and undermines potential regulatory frameworks, exactly because it restricts our theoretical understanding of volatile fields, like that of platform governance. As a result, we reckon that our analytical framework would greatly benefit from studying the space between governance and regulation, following thus the political sciences’ turn to these concepts ( Black, 2008; Braithwaite, 2011; Braithwaite *et al*., 2007). Perhaps, even more importantly, this would allow us to resituate the discussion around governance and broaden our analytical horizons. Consequently, we ought to combine regulation and governance as a theoretical framework to deepen our understanding of power relations in networked environments and their political economy.

### Governance

Having talked about regulation, we now turn our attention to governance. Some scholars study governance in two ways: either as an interchangeable term for regulation or as a way to describe an ecosystem from a structural or organisational standpoint ( Abbott & Snidal, 2009; Gjaltema *et al*., 2020). In this article, governance is understood as that politically charged notion that signifies “to govern” ( Gorwa, 2019b, p. 2). Governance, in this sense, possesses the attribute of authority that is tied with power, more akin to a Foucauldian interpretation as “the multiplicity of force relations immanent in the sphere in which they operate, and which constitute their own organization” ( Foucault, 1978, p. 92). Therefore, governance does not only have to do with the power of state over the public, as Foucault argued (ibid), but it is expanded to include the balance of power relations within a structured or networked space, like that of a market. Put simply, the power in “power relations,” that constitute governance, symbolises the interdependence, as well as the contentious interests among actors, which in turn, surface the “power plays” ( Carr, 2015) that irradiate the political economy of a given field.

Building upon the Foucauldian notion of “governmentality” ( *gouvernementalité*), which asks “how to govern” ( Foucault *et al*., 1991, p. 7), we could frame regulation as the mechanism for enforcing, preserving and/or expanding governance. This is where governance subtly differs from regulation. We could draw here an ontological parallel between this property of regulation and Foucault’s notion of government. Foucault argued that government refers to “the conduct of conduct” aiming to “shape, guide or affect the conduct of some person or persons” ( Gordon, 1991, p. 7). Therefore, we could claim that, governance shapes regulation directly (i.e., applying standards to a specific actor or cluster of actors) and/or indirectly (i.e., establishing and applying standards to the environment in which an actor is active; Koop & Lodge, 2015, p. 4). It seems, then, that there is a shared understanding of regulation’s *raison d’être* as a mechanism to alter behaviour (ibid, p. 5).

As hinted earlier, non-state actors have been increasingly taking up roles and responsibilities that were once exclusively held by the state, which has been progressively limited to a “regulatory state” ( Braithwaite, 2011), fuelling what some scholars have deemed as “regulatory capitalism” ( Braithwaite, 2008). Ever since the 1970s, with the Keynesian policies gradually falling apart in the Western world and the domination of neoliberalism ( Carr, 2015, p. 643; Foucault *et al*., 1991), state power has been dispensed to various non-state actors ( Majone, 1994; Mattli & Woods, 2009; Mazzucato, 2014). So, the current “networked governance” landscape ( Braithwaite *et al*., 2007; Drahos & Krygier, 2017) doesn’t facilitate top-down regulation, nor a traditional distinction between private and public actors. Concluding, we propose to define regulation as a governance mechanism, involving the intentional – direct or indirect - intervention in the activities of a stakeholder, with the intention to change that stakeholder’s modus operandi, which consequently has unpredictable consequences to the rest of the governance environment, given that governance is a dynamic and negotiable process.

### Regulation, governance, and polycentricity

Regulation and governance studies has recently emerged as an interdisciplinary field of scholarship which, as a founding principle, seeks to inform regulatory and law studies with the concept of governance ( Braithwaite *et al*., 2007). This is pursued by inviting scholars to study regulation in relation to its political and societal impact and, thus, steering us away from a narrower understanding of regulation as policy-making ( Koop & Lodge, 2015, p. 105). By following the paradigm of regulation and governance studies, we can better study regulation, as well as its political economy, because it allows us to consider the polycentric governance environments in which regulation is shaped and applied. These are environments which are characterised by “fragmentation, complexity and interdependence between actors, in which state and non-state actors are both regulators and regulated” ( Black, 2008).

As a result, we can imagine these multi-stakeholder environments ^1^ as contentious fora, where power relations among actors surface the interdependence of one another, while shaping the governing status quo. This consolidates the difference between regulation and governance, as well as their strong connection. Moreover, we argue that this also reminisces the “market place of ideas” ( Helberger, 2020), where the “bargaining” or “regulatory game” ( Abbott & Snidal, 2009, p. 48; Levi-Faur, 2011, p. 11) among stakeholders arguably promotes legitimacy and fairness through “radical pluralism” ( Cammaerts & Mansell, 2019).

However, as discussed earlier, this assertion can fall short as, more often than not, power asymmetries not only aren’t reduced, but they are also reinforced. Therefore, a reimagination of the way in which we study polycentricity is needed. As Carr acutely put it, “[the] more we understand about the opportunities and weaknesses of governance models for the internet (or anything else) the better equipped we are to effectively refine and amend those practices, functions and roles that comprise it” ( Carr, 2015, p. 643).

Julia Black contends that polycentric regimes are characterised by “fragmentation, complexity and interdependence between actors, in which state and non-state actors are both regulators and regulated” ( Koop & Lodge, 2015, p. 1). So, to design a best-practices approach to regulation, we ought first to be able to answer to questions of *legitimacy* and *accountability* in such an environment (ibid, p. 142). Accountability here is defined as “a particular type of relationship between different actors in which one gives account and another has the power or authority to impose consequences as a result” (ibid, p. 150). Additionally, legitimacy is defined as a social construct, providing an actor with “social credibility and acceptability” (ibid, p. 144).

Additionally, Black believes that the ability to structure specific narratives is a communication strategy deployed mainly by non-state actors, so as to enhance their legitimacy and affect accountability relationships (ibid, p. 151). She suggests three elements to be key in understanding the power relations at play within a polycentric regime: (i) the institutional environment in the construction of legitimacy; (ii) the dialectical nature of accountability relationships; (iii) and the communicative structures through which accountability occurs and legitimacy is constructed (ibid, p. 139).

However, Black purports that it is increasingly difficult to define who is to be held accountable at a given point in time, precisely due to the increased fragmentation of power (ibid, p. 139). Black structures her argumentation in regard to the regime’s accountability around a trilemma; if something goes wrong who do we hold to account: a single regulator (“one for all”), each decentralised regulator (“all for one”) or each actor individually (“each for itself”; Black, 2008, p. 143). Her position is somewhat of a hybrid, arguing that: “in order to assess the accountability of a regulatory regime […] the focus has to be on holding the outcomes of a regime as a whole accountable” (ibid, p. 157).

In other words, within a polycentric regime, we should be able to hold to account both each actor individually, as well as the regime collectively, in order to assess the effectiveness of regulation - or its lack thereof. Black’s approach, then, shows us how to better understand power relations among stakeholders, along with their “institutional embeddedness” (ibid, p. 157). This is made possible by homing in on accountability and legitimacy claims made to regulators, as well as the way in which they were responded to, so as to unearth the state of governance in an ecosystem.

As hinted in the introduction, Carr is one of the most critical voices in relevant literature concerning multi-stakeholderism ^2^. She essentially criticises what could be called a Habermasian obsession with normality based on rationality and consensus ( Cammaerts & Mansell, 2019; Davis, 2020). She criticises normative claims of “what the Internet ‘should be’” ( Carr, 2015, p. 642) for concealing their own agenda behind “widely resonant norms like ‘privacy’, ‘freedom’, ‘democracy’” (ibid). In addition, she has also criticised the lack of critical analysis of “multi-stakeholderism,” which she believes has “become almost synonymous with global Internet governance” (ibid, p. 641).

Of course, this does not condemn said notions but the way in which they are framed by specific stakeholders. Ultimately, Carr suggests that this normative interpretation leaves too little space for the expression of alternative views, as they are quickly shunned as opposition to those norms (ibid). She believes that the polycentric model has been so institutionally embedded, that it almost feels shielded by terms with “a strong normative component” such as “democracy promotion” or “Internet freedom” (ibid).

This theoretical approach comes with its own restrictions and biases. Carr’s take on the internet as “a mechanism for the projection of power” (ibid, p. 643) feels like a one-dimensional bashing on United States’ global interests in a post-Snowden world (ibid, p. 656). However, this should not reduce the argumentative power of her claim that, while the polycentric regime has been beneficial to the internet’s growth (ibid, p. 649), it has also been reinforcing and privileging existing power relations despite an ostensible decentralisation of power. As a result, she feels that there has not been enough space for critical voices to be heard, going as far as to suggest that “multi-stakeholderism [has] become a ‘rhetorical exercise aimed at neutralising criticism’ rather than a truly unique and participatory mechanism for governing a global resource” (ibid, p. 642).

Furthermore, Carr argues that there exist systemic issues of legitimacy and accountability with “rule-makers” and “rule-takers” (ibid, p. 640). She identifies three major stakeholders within this regime: *government*, *private sector*, and *civil society*. There seems to be a recurring *triadic model* within regulation and governance studies; Abbott and Snidal have named it the “governance triangle” (2009), which acts as a “heuristic device to structure analysis of widely varying forms of governance” (ibid, p. 52). According to the authors, this triangle consists of various zones, depending on the number of stakeholders involved in the deliberations, and each zone has a unique or mixed regulatory framework. Similarly, they also group actors in the same fashion as mentioned earlier: states, firms, and non-governmental organisations (NGOs).

However, we would be remiss not to highlight the consequences of discussing governance structures that refer only to these three actors: it normalises and reinforces a governance imaginary, where outsiders are excluded of the balance and, thus, risks replicating power imbalances and a quasi-elitist power structure. Additionally, the dynamics produced among these actors are contentious, which the authors often describe as “[a] transnational arena” or “bargaining game” ( Abbott & Snidal, 2009, p. 48), painting a picture of struggle for domination. Again, in a more Habermasian interpretation, the idealised exchange of rational arguments that, inevitably, will lead to a logical consensus ( Habermas, 1992), polycentric contention is framed as benign, constructing legitimacy ( Black, 2008) and fairness through “radical pluralism” ( Cammaerts & Mansell, 2019).

Yet, following Carr’s critique, we could argue that such an interpretation *neutralises* attempts to further politicise the discussion in regard to polycentricity, even if proponents argue that “[it celebrates] the inherently conflictual nature of ‘the political’” ( Cammaerts & Mansell, 2019, p. 5). Consequently, by adopting a more nuanced approach to mapping relevant stakeholders, we can better study the contested power relations that shape the political economy of platform governance and online content’s regulation.

## Framing platform governance and online content regulation

### Internet governance

The term “internet governance” dates back to the years after the commercial internet’s birth, circa mid 1990s; as Brousseau and Marzouki note, one of the earliest uses of internet governance, “as a tentative political construct” (2012, p. 2), was observed in the 1998 International Telecommunication conference. The reason why the authors label it as a political construct is because, up until that point, the term “internet governance,” was mostly related to technical issues of the internet, albeit a not well-known one. It was during that time that a specific socio-political agenda was also identified, along with its surrounding stakeholders ( Brousseau *et al*., 2012, p. 4). Certainly, even within those fora, actors could not entirely agree on the exact nature of participating stakeholders. Brousseau and Marzouki paint a picture of a dichotomy between the “technical community,” who were defensive of the internet’s principles and values that would be ensured by self-regulating institutions and the “civil society,” that identified social actors and “commonly defined rules” outside the strict “Internet community” as crucial (ibid).

A few years later, in 2006, the United Nations (UN) founded the Internet Governance Forum (IGF). This marked a new era for internet researchers and, largely, the internet’s modus operandi ( Hofmann *et al.*, 2016, p. 3). The IGF provided us with the first formal definition of internet governance: “Internet governance is the development and application by Governments, the private sector and civil society, in their respective roles, of shared principles, norms, rules, decision-making procedures and programmes that shape the evolution and use of the Internet.” This multi-stakeholder framing has truly been the cornerstone of internet research ever since. In this regard, we can discern the “manifestations of power and political values” ( Hofmann *et al*., 2016, p. 4) of participating actors colliding one with another, co-shaping governance, albeit rarely in an equal manner.

### Platform Governance and Online Content Regulation

More recently, there has been discussion concerning a new chapter in the multi-stakeholder internet governance model, that of online content regulation ( Douek, 2021) within what some have named the “platform governance” ( Gorwa, 2019b; Helberger, 2020). So, platform governance, at first glance, is a sub-governance field within the broader internet governance. The present internet governance status quo seems to be shifting away from a self-regulatory model to a rather collaborative one ( Gorwa, 2019b; Helberger, 2020). We reckon that the main reason behind this change, is the desire of private actors with strong “opinion power” ( Helberger, 2020) to ensure public legitimacy and fend off public intervention, thus, securing self-governance, that is the ability to function independently of public audit and accountability. Moreover, as Evelyn Douek sharply notes, “platforms […] [play] catch-up to societal demands for more responsible content moderation through self-regulatory innovations and reforms” (2021, p. 4; emphasis added).

Online content regulation is a useful rhetorical framework, that conceptually bridges content moderation and public regulation. Nevertheless, we should be wary of the context in which it is used: for instance, when Facebook publishes guidelines on online content regulation this should be seen as a move to formalise their content moderation process by welcoming collaborations with other stakeholders and, thus, mitigating part of their responsibilities. However, a problem that quickly arises with this approach, and which Anne Helberger hints at with the “opinion power” concept (2020), is that it obscures or, at least, downplays the governance conflicts, while putting too much faith on the amelioration of said “systems of content moderation.” Where internet governance was imagined to be a self-governed and self-regulated space, platform governance is imagined as a space of co-governance and co-regulation. Inviting co-governance and, consequently, co-regulation is, of course, not reproachable, quite the contrary; Douek believes that public regulation can make systems of content regulation “more accountable and credible” (2021, p. 59). However, we should also see such invitations as communication strategies aimed at building legitimacy and affecting accountability ( Black, 2008, p. 151)

There seems to be, then, a paradigmatic shift within internet governance regarding content moderation, that, as seen earlier, is by no means a new thing in the history of regulation: private actors invite other stakeholders to co-shape regulatory frameworks to co-exercise governance, thus dispersing regulatory risk and responsibility, while retaining clear governance boundaries. However, we argue that there is another layer to it: the underlying contention for cultural hegemony, a Gramscian concept that Carr applies to criticise the improvident adoption and appraisal of the internet governance scheme ( Carr, 2015). Cultural hegemony, as well as opinion power, that is real political power to influence democratic processes ( Helberger, 2020, p. 845), are inherently connected to polycentric governance regimes. We contend, then, that “platform governance” should be used as a useful theoretical model that *de facto* supports co-governance and, thus, allows for greater theorisation regarding the new chapter of online content regulation, that expands the “governance triangle” ( Abbott & Snidal, 2009; Gorwa, 2019a); hence, it is not so much of a sub-field of internet governance but rather its evolution.

So, while the governance triangle serves as a valuable conceptual model of pinpointing stakeholders, it restrains us from having a more nuanced picture. However, it should be mentioned that Roberta Gorwa’s re-framing of the triangle to illustrate the European content regulation landscape ( Gorwa, 2019a, p. 7), adds some nuance concerning the stakeholders’ relations concerning online platforms’ regulation. Nonetheless, we argue that there are some stakeholders that are difficult to group together and that clustering them solely based on a ‘spatial’ manner (i.e., where they stand in the governance triangle) does not do enough justice to their unique nature. For instance, news media (i.e., newspapers, broadcast stations, digital native news media, etc.) might fall in the same category as digital platforms, given that they are both private actors, but their interest and power differ vastly.

**Figure 1.**
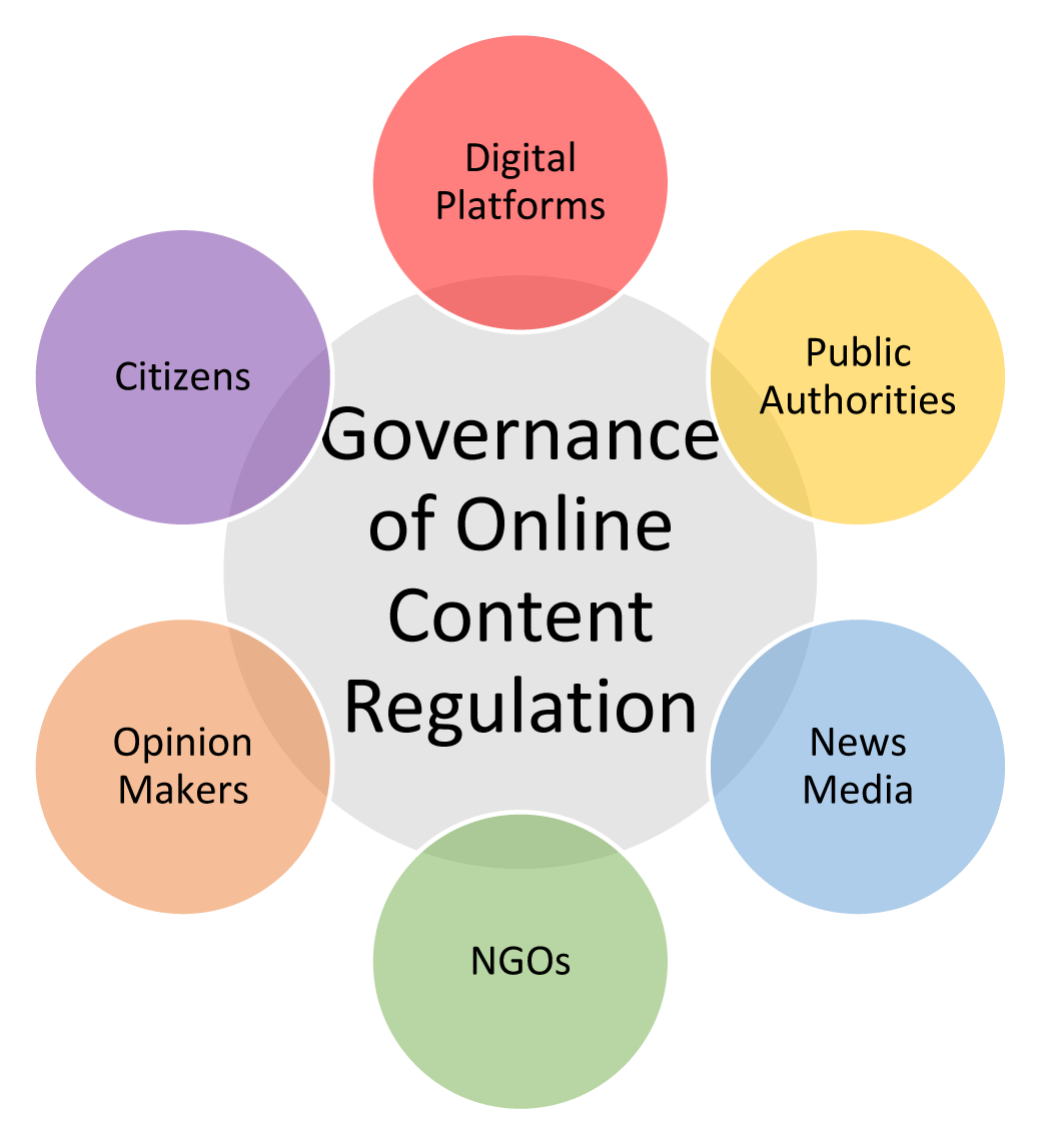
The Governance of Online Content Regulation.

As a result, we propose that a more appropriate concept would be that of “governance clusters,” ( Figure 1) which are comprised of actors sharing some common fundamental principles and interests. A working typology of platform governance clusters could be the following: (i) digital platforms, (ii) public authorities, (iii) non-governmental organisations, (iv) news media, (v) citizens, and, lastly, (vi) opinion makers. So, instead of trying to adjust the governance triangle model, we propose the depiction of these clusters as a circle that contains smaller intercommunicating circles.

i. **Digital platforms**, while will not be discussed in depth here, refer to social infomediaries ( Smyrnaios & Rebillard, 2019) and social media platforms that host, curate and disseminate content online.
ii. **Public authorities** refer to public actors, like governmental, national or supranational organisations, who are either elected or appointed by elected officials, with the authority to pass regulations, policies or legislation.
iii. **NGOs** might refer to civil society organisations, workers’ associations or platforms’ lobby groups, which are meant to promote their partners’ interests and deliberate with other stakeholders regarding regulatory frameworks.
iv. The cluster of **news media** refers primarily to news organisations that play an active role in shaping the regulatory agenda of online content. Platforms might argue that news’ revenue is “ minimal,” but their role in platform governance is crucial ( Napoli, 2015; Smyrnaios & Rebillard, 2019) because, among others, they make platforms nodes of public interest, where information is centralised ( Helmond, 2015). Additionally, ever since the consolidation of online platforms, news organisations have been trying to stay afloat and retain or increase their visibility. To that end, many news organisations have struck different deals with online platforms, while others have been pushing their associations to either collectively negotiate with platforms or push public authorities to intervene.
v. The fifth cluster refers to **citizens**, who are theoretically represented in the governance deliberations by civil society organisations ( Regilme, 2018). However, in modern deliberations, we see citizens participating individually: for instance, the European Commission has put public, open consultations in place, where every stakeholder can participate to help officials draft regulations. Furthermore, it is generally claimed that civil society organisations, theoretically, exist to represent citizens and the public in multi-stakeholder deliberations; yet, we believe that this is more of a hypothesis rather than an axiom, thus, public consensus might be nothing more than wishful thinking ( Flyvbjerg, 1998, pp. 214, 229)?
vi. Last, **opinion makers** ^3^, like academics, have turned out to be crucial in deliberating with policymakers or civil society organisations or, even, explaining complicated issues to the public.

A recent example could help us present how our conceptual framework could be used to study platform governance. Recently, Australia proposed the introduction of a new media law, which would oblige major social media platforms and, specifically, Facebook and Google, to pay publishers for news content hosted by their services. Google immediately reacted and sought to close deals with numerous publishers, mostly with major ones, while Facebook in an unprecedented move pulled the plug on news sharing, blocking even governmental agencies that were informing the public regarding the development of the coronavirus disease 2019 (COVID-19) pandemic. Subsequently, the Australian government directly negotiated with Facebook and after some amendments to the proposed law, the social media platform re-activated the news sharing on their service and appraised the changes.

So, the Australian government attempted to proceed with top-down regulation, or what Levi-Faur defines as “enforced self-regulation” (p. 11), as the bill would not impose specific tariffs to be paid but rather oblige platforms to negotiate deals with news publishers, and only if a deal wasn’t possible would the government intervene through arbitration. After Facebook’s unilateral blocking of access to content, we could say that the Australian government was forced to co-shaping the regulatory framework. In addition, this contention between Australia and social media platforms triggered a chain of reactions. For example, shortly after, Microsoft joined forces with some European news publishers to, first, criticise Facebook and Google and, then, to announce a project that will aim to “develop a legal solution to ‘mandate payments’ for the use of content by ‘gatekeepers’ that have dominant market power.” This development highlights, on the one hand, the increasing role that the governance cluster of news organisations plays and, on the other, the promotion of co-regulation by consolidated private actors as a means to promote vested rather than public interests.

## Conclusion

This paper sought out to introduce a comprehensive yet non-exhaustive overview of literature concerning the concepts of regulation and governance, as well as to connect them to the emerging scholarship engaging with social media platforms. Specifically, the paper introduced the various approaches to defining regulation and governance in tandem with polycentricity, and then proceeded with connecting said notions, primarily, with two new trending research fields: platform governance and online content regulation. To recapitulate, we define regulation as a governance mechanism, involving the intentional – direct or indirect - intervention in the activities of a stakeholder, with the intention to change that stakeholder’s modus operandi, which consequently has unpredictable consequences to the rest of the governance environment. Moreover, where traditional governance literature conceptualised stakeholders as a triangle, we propose imagining them as overlapping circles of governance clusters with competing interests, going beyond the triad of state, firm, and NGOs.

Also, the article briefly touched upon internet governance and provided a synopsis of the critique surrounding its governance model and, mainly, the multi-stakeholder regime. The key takeaway point is that it has monopolised scholars and policymakers so much so that any critique towards the model is perceived as an attack to democracy or plurality ( Carr, 2015), which has led to the weakening of critical analyses and has perpetuated power asymmetries. In contrast, we argue that there is a timely need to reimagine conceptual models of governance in order to reinforce polycentricity and decentralisation.

We contend that such reimagination is possible by studying platform governance as a de facto co-governance model, instead of the self-governance model first celebrated by internet governance. As a result, the starting ground is different; yet, we ought to be wary of the risk of replicating the internet governance’s status quo. In other words, we propose to study the governance of online content and social media platforms not as a sub-section of internet governance but as a conceptual evolution with existential stakes.

Future research should look into studying the new governance clusters in concert to developments in the online content regulation front. For instance, citizens’ contributions to governance deliberations through the European Commission’s public consultations or academics’ participation in panels aimed at designing or assessing regulatory frameworks. In addition, much more detailed work is needed to theoretically underpin the emerging field of platform governance, while a systematic literature review could certainly help us understand when and how the term gained traction. As hinted throughout the paper, platform governance can be an illuminating conceptual vehicle to explore the implications of online content regulation and the governance deliberations to the public discourse and the public sphere in general ( Papacharissi, 2002; Salikov, 2018).

## Data availability

All data underlying the results are available as part of the article and no additional source data are required.
