# The existential stakes of platform governance: a critical literature review

**DOI:** 10.12688/openreseurope.13358.2

**Published:** 2021-07-01

**Authors:** Charilaos Papaevangelou

**Affiliations:** 1 JOLT-ETN / LERASS, University Paul Sabatier - Toulouse III, Toulouse, France

**Keywords:** Platform governance, online content regulation, multi-stakeholder governance, social media regulation, regulatory governance

## Abstract

This study introduces a comprehensive overview of literature concerning the concepts of regulation and governance, and attempts to connect them to scholarly works that deal with the governance of and by social media platforms. The paper provides fundamental definitions of regulation and governance, along with a critique of polycentricity or multi-stakeholderism, in order to contextualise the discussion around platform governance and, subsequently, online content regulation. Moreover, where traditional governance literature conceptualised stakeholders as a triangle, this article proposes going beyond the triad of public, private and non-governmental actors, to account for previously invisible stakeholder clusters, like citizens and news media organisations. This paper also contends that, while platform governance is an important field of study and practice, the way it has been structured and investigated so far, is posing an existential risk to the broader internet governance structure, primarily, because of the danger of conflating the internet with platforms. As a result, there exists a timely need to reimagine the way in which we understand and study phenomena related to platform governance by adjusting our conceptual and analytical heuristics. So, this article wishes to expand the theorisation of this field in order to better engage with complicated platform governance issues, like the development of regulatory frameworks concerning online content regulation.

## Plain language summary

This article examines academic literature regarding the notions of regulation and governance, trying to define what they mean, how they are used depending on the field of application and how they are framed specifically when studying online platforms (e.g., Facebook, Google, etc.). The author begins by acknowledging that, while this is not an exhaustive research, there has been a wide embrace of terms like “platform governance” and “online content regulation,” even by policymakers. Therefore, the author is interested in defining what these concepts mean and how they can be used to study online platforms. The author also provides a brief historical retrospective on how academics have studied the way that the internet is structured and governed by participating stakeholders. Additionally, the author believes that, whereas in the early 1990s cyber-utopians imagined the internet to be a democratised, decentralised and self-regulated space, away from state interventions, we are now in the age of platform governance. Platform governance is a term inherently connected with the multiplicity and plurality of stakeholders but places online platforms at the epicentre. This is quite useful because it allows us to better engage with platforms and, specifically, social media and infomediaries. So, where internet governance began by celebrating independence, platform governance begins by celebrating collaborations with a myriad of stakeholders, including states. Finally, the paper argues that by being able to discern these notions, as well as explore as many stakeholders as possible, we are better equipped to reimagine how regulatory frameworks should be designed, especially those that are related to online content, which constitutes a large part of our online discussions on social media and elsewhere. Last, this work also wishes to contribute to the expansion of the discussion concerning the regulation of online platforms to include the participation of citizens and users.

## Introduction

Recently, a significant volume of scholarly work has embraced the burgeoning use of notions like *platform governance* ( Caplan & Gillespie, 2020; Fan & Zhang, 2020; Gorwa, 2019b; Haggart, 2020; Mazzucato *et al.*, 2020; Napoli, 2015) and *online content regulation* ( Douek, 2021; Gorwa, 2019a; Land, 2019). This has further expanded the interdisciplinary boundaries of literature that relate to regulation and governance, and has effectively consolidated the concept. The underlying common ground of these works is online platforms and, specifically, social media platforms ( Bucher & Helmond, 2018; Gillespie, 2010; Smyrnaios & Rebillard, 2019). This paper seeks to theoretically frame the discussion with works stemming from the broader field of regulation and governance ( Braithwaite *et al.*, 2007; Kjaer & Vetterlein, 2018; Levi-Faur, 2011), media studies ( Flew *et al.*, 2021; Napoli, 2015; Puppis, 2010), internet governance ( DeNardis, 2020; Hofmann *et al.*, 2016) and research studying content moderation or regulation of social media platforms ( Douek, 2019; Flew *et al.*, 2019; Gorwa, 2019a). Therefore, this paper serves as a critical exploration of relevant literature, aspiring primarily to help online media scholars to navigate the multifaceted domain of platform regulation.

In this paper, regulation is defined as a governance mechanism, involving the intentional – direct or indirect - intervention in the activities of a stakeholder, with the intention to change a stakeholder’s modus operandi, which, in turn, may have unpredictable – yet measurable - consequences to the governance regime ( Black, 2008; Koop & Lodge, 2015; Levi-Faur, 2011). For reasons of clarity, governance is defined here as a complex networked structure that accommodates different stakeholders, who are connected to and coordinated through various types of regulations, norms and practices ( Héritier, 2002; Offe, 2009; Stoker, 1998). As can be inferred, there is an innate connection between regulation and governance insofar as governance offers the structure in which political-economic relations formulate, primarily, through regulation.

What is more, this paper approaches governance as a bifold concept: governance as *structure* and governance as *power*. The former allows us to examine an ecosystem from a structural standpoint and map its stakeholders, whereas the latter accounts for governance’s analytical element that allows us to inquire an ecosystem’s power relations through their (in)formal arrangements and deliberations or produced regulatory frameworks ( Gjaltema *et al.,* 2020; Mattli & Woods, 2009).

The works considered here are meant to be representative of relevant literature and their selection was done by selecting key publications connected with the topics of platform governance, as well as works that have studied regulation and governance This article is by no means an exhaustive piece of research, but rather it is an invitation to investigate the interdisciplinarity and depths of a vibrant field, which seeks to understand the governance of online platforms and their regulation, as well as their implications relating to democracy and public discourse. This is an important field, not only because it expands our research horizons, but also because it aims to inform stakeholders found at every position within the governance spectrum. It is thus a timely effort to properly situate the discussion revolving around platform governance and propose a new way to theorise about regulations that aim to tackle complex platform-related issues, such as content moderation.

The paper is structured in the following way: the first section introduces the discussion around regulation and governance, the second section zooms in on polycentric or multi-stakeholder governance regimes, and the final part attempts to propose an operational framework to better study platform governance to a) consider actors that do not fall in traditional categories (i.e., state, firm, NGO), b) consider inter-stakeholder competing interests, and c) consider the importance of citizens and users in modern networked structures. As this is part of a long-term research project, it is bound to change over time; however, the benefit of the proposed operationalisation is adjustability to the deployed field of inquiry.

Finally, the literature review presented here, attempts to surface an existential risk that lies with the way that current scholarship approaches platform regulation and governance: that of conflating the internet with large social media platforms. Therefore, it is also this paper’s goal to discern the two so as to study platform governance as a distinct field from that of the broader internet governance structure and contribute to a much-needed reimagination of the way that regulatory frameworks are developed within current governance structures.

## Towards a definition of regulation and governance

### Regulation

Regulation consists of a large gamut of factors, including “politics, policies, institutions and effectiveness of formal and informal controls” ( Levi-Faur, 2011, p. 16); in other words, to study regulation, one has to take into consideration a plethora of elements, alongside their innate political and, often, conflictual attributes. David-Levi Faur offers us a comprehensive overview of the multidisciplinary field of regulation in his seminal book *Handbook on the Politics of Regulation* (2011), inviting us to consider how regulation’s meaning can change depending on the field of employment.

For instance, regulation has become a distinct field of international practice and research, especially after the introduction of the economic theory of regulation ( Stigler, 1971). Certainly, the definition of regulation varies even among economic theorists: some argue that it acts as another weapon of neoliberalism against the welfare state ( Majone, 1994), while others believe it to be an important tool to fuel competition ( Levi-Faur, 2011, p. 3).

In any case, the concept of regulation expands well beyond the theory of economy and covers the field of standard-setting, administration and, more broadly, the power of institutions. Some scholars have talked about the benefits of regulation against consumer exploitation, environmental misdoings and other activities in a rather pragmatistic way ( Koop & Lodge, 2015; Marx, 2011). Moreover, one could not neglect adding to this long interdisciplinary list, the framing of regulation by social and political sciences as a means of control ( Beresford, 2003; Levi-Faur, 2011, pp. 3, 16) that, inter alia, seeks to dictate a change in behaviour ( Koop & Lodge, 2015).

Levi-Faur frames it as “the *ex-ante* bureaucratic legalisation of prescriptive rules and the monitoring and enforcement of these rules by social, business, and political actors on other social, business, and political actors” ( Levi-Faur, 2011, p. 6; emphasis theirs). Consequently, this is a definition with a distinct organisational approach to regulation, while excluding the “legislative or judicial rule making” (ibid). In other words, Leiv-Faur describes a co-regulatory framework, in which the state sets rules, that are then monitored and enforced through the collaboration of social, business, and political actors. Elsewhere, Koop and Lodge frame regulation as following: "[it is the] intentional intervention in the activities of a target population, where the intervention is typically direct – involving binding standard-setting, monitoring, and sanctioning – and exercised by public-sector actors on the [activities] of private-sector actors" ( Koop & Lodge, 2015, p. 106). The two definitions share the same characteristics concerning how regulation works (i.e., standard-setting and not rule-making, monitoring and enforcement) and allow us to consider, on the one hand, the collaborative nature of regulation (i.e., multi-stakeholderism) and, on the other hand, its pre-emptive aspect meant to control behaviour.

In addition, one other significant common point of the definitions is the development of targeted and binding rules, which Black purports aim to “change the behaviour of others […] through a combination of rules and norms” ( Black, 2008, p. 139). As a result, we can further distinguish regulation according to its end-goals,. So, on the one part, there is regulation that serves the “public interest” ( Hofmann *et al.*, 2016, p. 1410; Levi-Faur, 2011, p. 28) and, on the other, regulation that “mainly serves private interests,” which some have called “deregulation” ( Levi-Faur, 2011, p. 28). It is made, thus, visible that the envisioned goal of regulation as beneficial to the public interest is by no means a given; it is hard to argue that all actors in a competing environment share the same values. It should be also noted that scholars of social media platforms have been approaching regulation with a “public-interest” approach, following the long tradition of media and journalism ( Napoli, 2015; van Dijck *et al.*, 2018).

This is why it is very important to acknowledge that regulation is itself a product of negotiations and power dynamics. Therefore, while regulation concerns primarily ex-ante standard-setting or rules, it may be possible to predict a regulation’s outcomes (e.g., what type of content will be deemed illegal or violating a platform’s policy) but its long-term effects on governance are unpredictable. To this end, some argue that the key way of mitigating such regulatory risk is the multi-stakeholder governance model ( Black, 2008). In other words, regulation that is developed by a single authority with specific results in mind is less flexible and, thus, less effective when dealing with ever-everchanging phenomena; hence, polycentricity is often framed as panacea, which has come to monopolise the way of analytically framing the discussion revolving around governance ( Carr, 2015; Hofmann, 2020). At any rate, as relevant literature attests, all governance structures include a multitude of different stakeholders deliberating regulatory frameworks, which has accelerated the decentralisation of state power, yet has exacerbated the complexity of governance regimes ( Abbott & Snidal, 2009; Bernstein & Cashore, 2007; Büthe & Mattli, 2011; Levi-Faur, 2011; Majone, 1994).

Furthermore, the actors most commonly found within these power structures are: state actors, non-state or market actors, and non-governmental or civil actors ( Abbott & Snidal, 2009, pp. 8–10; Gorwa, 2019a, p. 2; Levi-Faur, 2011, p. 10). Accordingly, three types of regulation are most commonly met in the relevant literature: self-regulation, co-regulation, and top-down (or ‘command-and-control’) regulation ( Gorwa, 2019b, p. 853; Hirsch, 2013; Levi-Faur, 2011, p. 531; Marsden, 2011, pp. 13–14):

• **Self-regulation**: This type of regulation refers primarily to non-state, “voluntary and ‘non-binding’” agreements and principles ( Gorwa, 2019b), such as platforms’ “Terms of Services” ( Bietti, 2020; Suzor, 2019) or self-organised industry groups, such as the “Global Internet Forum to Counter Terrorism” ( Gorwa, 2019b). This type of regulation is by and large preferred by firms as it greatly reduces costs of implementing formal legislation ( Mattli & Woods, 2009, p. 8), which has also given way to the privatization of regulation ( Büthe & Mattli, 2011). Moreover, this type of regulation has little legitimacy in polycentric regimes, as it is tied to a *laissez-faire* attitude ( Bernstein & Cashore, 2007; Büthe & Mattli, 2011; Flew *et al.*, 2019; Marsden, 2011), which often lacks legal repercussions. Moreover, self-regulation seeks to consolidate an actor’s (or a cluster of actors) self-governance, that is, their independence of a hierarchically higher authority to hold them to account.

• **Co-regulation**: This type of regulation primarily refers to the attempt of combining the ‘best’ of all three actors’ competencies, which Abbott and Snidal argue are: “interdependence, representativeness, expertise, and operational capacity” (p. 66). We could argue that this type of regulation acts as the cornerstone of the polycentric regime and is thus often depicted as essential to democratic representation and plurality ( Black, 2008; Cammaerts & Mansell, 2019). However, each actor has its own agenda, making contention unavoidable. A large number of scholars, policymakers and, recently, online platforms, are in favour of this type of regulation, also called as “soft-law” ( Mattli & Woods, 2009, p. 1), because it “[opens up a] more interesting [conversation] than a static no-regulation versus state regulation binary choice” ( Marsden, 2011, p. 242). Co-regulation seeks to consolidate a shared governance (co-governance) among stakeholders. Accountability here varies but, in most cases, it takes the shape of periodic transparency reports, audits, and repercussions in cases where notice isn’t followed by action.

• **Top-down regulation**: Last, self-regulation refers to state regulation, which is usually passed by public authorities in the form of official legislation, or “hard rules” ( Mattli & Woods, 2009, p. 1), often directly intervening in an industry or a market. State regulation is usually critiqued as cumbersome and counterproductive, especially concerning innovation ( Bostoen, 2018). However, it can work as the “baseline” ( Gorwa, 2019b, p. 8) upon which other types of regulation are built, “either as complements to fill in certain gaps, or as substitutes to proposals perceived as overly invasive or harmful to human rights” (ibid). Its legitimacy can vary depending on the state which regulates and the political state of affairs (e.g., democratic processes, political representation, etc.). Accountability is high because there are legal consequences to actors who do not abide by the state’s regulation and it is the state that will hold to account a rogue actor. However, it should be noted that this too is to be taken with a grain of salt because, on the one hand, the state has its own agenda (e.g., to satisfy electorates) and, on the other, because the state itself might avoid accountability due to authoritative concentration of power.

Levi-Faur adds some nuances to the traditional typology: according to him, “pure self-regulation” (p. 531) is a branch of “[hybrid] meta-regulation,” which refers to a confined role of the regulator to the “institutionalisation and monitoring” of standards and rules (p. 11). He also adds another type of regulation, that of “[hybrid] multi-level regulation,” emphasising the geopolitical implications of regulators, where the “regulatory authority is allocated to different levels of territorial tiers” (ibid). This paper contends that while the latter may add an important nuance to critical analyses, the former rather complexifies the discussion; conversely, we propose restricting meta-regulation to that, which “regulates any other form of regulation” (Parker in Levi-Faur, 2011, p. 11). In any case, by going through the above-mentioned typology of regulations, it can be made clear that the concept of regulation is inherently tied to the notion of governance; this is because regulation is, in and of itself, an exercise of authority and power aiming to shape governmental structures ( Kjaer & Vetterlein, 2018).

As a result, many scholars have been increasingly treating regulation and governance almost synonymously ( Hofmann *et al.*, 2016), while some have been talking about “regulatory governance” ( Kjaer & Vetterlein, 2018): that is, “governance through regulation” (ibid, p. 499). However, it is not entirely sure as to why develop the concept of “regulatory governance,” given that modern multi-stakeholder governance regimes implicitly involve structuring power relations through regulation – or its lack thereof. Nevertheless, Kjaer and Vetterlein provide a detailed and convincing account of the historical aspect of regulation as a governance praxis, which is in line with this paper’s framing.

Subsequently, in the next section we will cover governance. We should first underline that regulation and governance are not synonymous; treating them as such “[strips regulation] of some analytical potential” (p. 6) and undermines potential regulatory frameworks, exactly because it restricts our theoretical understanding of volatile fields, like that of platform governance. As a result, this paper studies the space between governance and regulation, following thus the political sciences’ turn to these concepts ( Black, 2008; Braithwaite, 2011; Braithwaite *et al.*, 2007). Perhaps, even more importantly, this would allow us to resituate the discussion around governance and broaden our analytical horizons. Consequently, we ought to combine regulation and governance as a theoretical framework to deepen our understanding of power relations in networked environments and, even, their political economy.

Concluding, the paper proposes to define regulation as a governance mechanism, involving the intentional – direct (i.e., applying standards to a specific actor or cluster of actors) or indirect (i.e., establishing and applying standards to the environment in which an actor is active; Koop & Lodge, 2015, p. 4)- intervention in the activities of a stakeholder, with the intention to change that stakeholder’s modus operandi, which consequently has unpredictable consequences to the rest of the governance environment, given that governance is a dynamic and negotiable process.

### Governance

As mentioned in the introduction, this paper takes into account the bifold nature of governance: both as a concept used to describe the shift from one authoritative source of power (i.e., the state) to a multiplicity of stakeholders and as an analytical vehicle to analyse power relations of these stakeholders. While the root of governance can be found at the science of new institutional economics ( Bulmer, 1998; Puppis, 2010, p. 135), this paper is predominantly interested in its development by social and political scientists. As such, in this article, governance is primarily understood as that politically charged notion that signifies “to govern” ( Gorwa, 2019b, p. 2) and, specifically, to govern through regulation.

Governance, in this sense, possesses the attribute of authority that is tied with power, more akin to a Foucauldian interpretation as “the multiplicity of force relations immanent in the sphere in which they operate, and which constitute their own organization” ( Foucault, 1978, p. 92). Therefore, governance does not only have to do with the power of state over the public, as Foucault argued (ibid), but it is expanded to include the balance of relations within a structured or networked space. Put simply, the power in “power relations,” that constitute governance, symbolises the interdependence, as well as the contentious interests among actors, which in turn, surface the “power plays” ( Carr, 2015) that irradiate the political economy of a given field.

Furthermore, governance, has been extensively studied in tandem with international relations, as the movement of globalisation claimed a significant part of governmental, that is, state power ( Kjaer & Vetterlein, 2018, p. 500). In his paper on media governance, Puppis reviews relevant political science literature and suggests that exist two approaches to defining governance: a narrower and a broader ( Puppis, 2010).

According to the narrower approach, governance is framed as a complex and multi-layered network of power relations among various stakeholders, “creating the conditions for ordered rule and collective action” ( Stoker, 1998, p. 17). Subsequently, here, governance marks the shift from government “to a new process of governing” ( Rhodes, 1996, pp. 652–653), where the state is restricted to “political steering” of “non-hierarchical” governance structures ( Héritier, 2002, p. 2, as cited in Puppis, 2010, p. 137). Additionally, according to the broader approach, governance “[goes] beyond so-called new forms of regulation and [focuses] on collective coordination (emphasis theirs) in general” ( Puppis, 2010, p. 137). Consequently, the broader approach to defining governance takes into account the role of the state and involves “a mix of governing efforts by public and private actors occurring at different levels and in different modes” ( Kooiman, 2003, p. 3). As a result, in this approach, the state is replaced as the central authoritative node with “a multiplicity of governing and each other influencing actors” (Kooiman & Van Vliet, 1993, p. 64, as cited in Stoker, 1998, p.17) but still holds its “monopoly on the legitimate use of coercion” and regulatory intervention ( Black, 2002; Puppis, 2010, p. 137).

I argue that these two approaches are not oppositional one to another but rather highlight different aspects of governance. For instance, those who attempt to narrowly define governance as the new model of “governing without government” ( Rhodes, 1996) may – inadvertently - promulgate the neoliberal “minimal state” paradigm (Bevir, 2009, p. 5, as cited in Puppis, 2010, p. 137), in which the state is limited to a managerial position and several of its functions are outsourced to the private sector ( Büthe & Mattli, 2011; Rhodes, 1996; Rosenau, 1992). As such, it seems that both approaches understand that governance signifies that “boundaries between and within public and private sectors have become blurred” ( Stoker, 1998, p. 17), but differ primarily in assessing the state’s role.

According to the Foucauldian notion of “governmentality” ( *gouvernementalité*), which asks “how to govern” ( Foucault *et al.*, 1991, p. 7), we could frame all forms of regulation as the mechanism for enforcing, preserving and/or expanding governance. We could draw here an ontological parallel between this property of regulation and Foucault’s notion of government. Foucault argued that government refers to “the conduct of conduct” aiming to “shape, guide or affect the conduct of some person or persons” ( Gordon, 1991, p. 7). It seems, then, that there is a shared understanding of regulation’s *raison d’être* as a mechanism to alter behaviour (ibid, p. 5).

As hinted earlier, non-state actors have been increasingly taking up roles and responsibilities that were once exclusively held by the state, which has been progressively limited to a “regulatory state” ( Braithwaite, 2011), fuelling what some scholars have deemed as “regulatory capitalism” ( Braithwaite, 2008). Ever since the 1970s, with the Keynesian policies gradually falling apart in the Western world and the domination of neoliberalism ( Carr, 2015, p. 643; Foucault *et al.*, 1991), state power has been dispensed to various non-state actors ( Majone, 1994; Mattli & Woods, 2009; Mazzucato, 2014). So, the current “networked governance” landscape ( Braithwaite *et al.*, 2007; Drahos & Krygier, 2017) does not easily allow for top-down regulation, nor a traditional distinction between private and public actors.

To summarise, this paper approaches the definition of governance in two interrelated ways: *governance as structure* and *governance as power*. The former refers to governance as a complex networked structure that accommodates different stakeholders that are connected and coordinated through various types of regulations, norms and practices, whereas the latter refers to governance as a politically charged notion that allow for power relations among governance stakeholders to formulate, primarily, through regulations. What is more, these two approaches hold different analytical purposes: the former is used as a way to conceptualise governance structures based on its outcomes (e.g., Abbott and Snidal’s “Governance Triangle”), whereas the latter is used as a way to study the procedures that form the stakeholders’ power relations (e.g., how a regulation was formed).

### Regulation, governance, and multi-stakeholderism

Regulation and governance studies has recently emerged as an interdisciplinary field of scholarship which, as a founding principle, seeks to inform regulatory and law studies with the concept of governance ( Braithwaite *et al.*, 2007). This is pursued by inviting scholars to study regulation in relation to its political and societal impact and, thus, steering us away from a narrower understanding of regulation as policy-making ( Koop & Lodge, 2015, p. 105). By looking regulation in relation to governance, we can better study the polycentric governance environments in which regulation is shaped and applied. These are environments which are characterised by “fragmentation, complexity and interdependence between actors, in which state and non-state actors are both regulators and regulated” ( Black, 2008; Koop & Lodge, 2015, p. 1)

As a result, these multi-stakeholder environments ^1^ are contentious fora, where power relations among actors surface the interdependence of one another, while shaping the governing status quo, which is “most likely to promote their own interests” ( Carr, 2015, p. 645). Moreover, this situation also reinforces the “radical pluralist” ( Cammaerts & Mansell, 2019) criticism of the consensual “market place of ideas” ( Helberger, 2020), insofar as the “bargaining” or “regulatory game” ( Abbott & Snidal, 2009, p. 48; Levi-Faur, 2011, p. 11) among stakeholders does not necessarily promote legitimacy and fairness but may perpetuate existing power relations.

However, as discussed earlier, this assertion can fall short as, more often than not, power asymmetries not only aren’t reduced, but they are also reinforced. Therefore, a reimagination of the way in which we study multi-stakeholderism is needed. Interestingly, the term “multistakeholder” first emerged in the 1990s and was officially used in the context of the internet with the establishment of the Working Group on Internet Governance (WGIG) ( Hofmann, 2020, p. 256; Palladino & Santaniello, 2021). In any case, as Carr acutely put it, “[the] more we understand about the opportunities and weaknesses of governance models for the internet (or anything else) the better equipped we are to effectively refine and amend those practices, functions and roles that comprise it” ( Carr, 2015, p. 643).

So, to study platform governance and, consequently, platform regulation, we ought first to define two core elements that are often at the heart of regulatory frameworks: *legitimacy* and *accountability* ( Carr, 2015, p. 142). By assessing a governance regime’s legitimacy and accountability, along with its constituents’ efforts to reify these two fundamental elements, we can infer critical results of that regime’s status quo and power relations.

Accountability here is defined as “a particular type of relationship between different actors in which one gives account and another has the power or authority to impose consequences as a result” (ibid, p. 150). However, Black purports that it is increasingly difficult to define who is to be held accountable at a given point in time, precisely due to the increased fragmentation of power ( Black, 2008, p. 139). Black structures her argumentation in regard to the regime’s accountability around a trilemma; if something goes wrong, who do we hold to account: a single regulator (“one for all”), each decentralised regulator (“all for one”) or each actor individually (“each for itself”; Black, 2008, p. 143). Her position is somewhat of a hybrid, arguing that: “in order to assess the accountability of a regulatory regime […] the focus has to be on holding the outcomes of a regime as a whole accountable” (ibid, p. 157).

In other words, within a polycentric regime, we should be able to hold to account both each actor individually, as well as the regime collectively, in order to assess the effectiveness of regulation - or its lack thereof. Black’s approach, then, shows us how to better understand power relations among stakeholders, along with their “institutional embeddedness” (ibid, p. 157). This is made possible by homing in on accountability and legitimacy claims made to regulators, as well as the way in which they were responded to, so as to unearth the state of governance in an ecosystem.

Additionally, legitimacy is defined as a social construct, providing an actor with “social credibility and acceptability” (ibid, p. 144). Santaniello and Palladino offer a valuable historical overview of the scholarship around legitimacy and, specifically, talk about two perspectives of legitimacy: a normative and a descriptive ( Palladino & Santaniello, 2021, p. 31). The former, they argue, effectively looks at an actor’s “values and principles,” which fuel its “right to rule” (i.e., rule-makers), whereas the latter examines the “audiences and their reasons to believe that an authority is appropriate” (i.e., rule-takers; ibid). Therefore, legitimacy is essentially an expansion or evidence of power and a key to assuming authority in a governance regime.

Provided that the internet, as we’ll see in more detail later, is inherently tied to the concept of multi-stakeholderism, gauging legitimacy, as with accountability, is tremendously difficult. There is no single one authority that rules the internet. As a result, many scholars have argued that legitimacy in these environments is primarily related to the participation of a plethora of actors ( Flew *et al.*, 2019; Haggart & Keller, 2021; Palladino & Santaniello, 2021, p. 32). Consequently, a governance regime’s legitimacy is depended on its openness and inclusiveness, so that “all the categories of actors affected by a particular issue were involved in the decision-making process” ( Palladino & Santaniello, 2021, p. 33).

As hinted in the introduction, Carr is one of the most critical voices in relevant literature concerning multi-stakeholderism. She essentially criticises what could be called a Habermasian obsession with normality based on rationality and consensus ( Cammaerts & Mansell, 2019; Davis, 2020). She criticises normative claims of “what the Internet ‘should be’” ( Carr, 2015, p. 642) for concealing their own agenda behind “widely resonant norms like ‘privacy’, ‘freedom’, ‘democracy’” (ibid). In addition, she has also criticised the lack of critical analysis of “multi-stakeholderism,” which she believes has “become almost synonymous with global Internet governance” (ibid, p. 641).

Of course, this does not condemn said notions but the way in which they are framed by specific stakeholders. Ultimately, Carr suggests that this normative interpretation leaves too little space for the expression of alternative views, as they are quickly shunned as opposition to those norms (ibid). She believes that the polycentric model has been so institutionally embedded, that it almost feels shielded by terms with “a strong normative component” such as “democracy promotion” or “Internet freedom” (ibid).

This theoretical approach comes with its own restrictions and biases. Carr’s take on the internet as “a mechanism for the projection of power” (ibid, p. 643) feels like a one-dimensional bashing on United States’ global interests in a post-Snowden world (ibid, p. 656). However, this should not reduce the argumentative power of her claim that, while the polycentric regime has been beneficial to the internet’s growth (ibid, p. 649), it has also been reinforcing and privileging existing power relations despite an ostensible decentralisation of power. As a result, she feels that there has not been enough space for critical voices to be heard, going as far as to suggest that “multi-stakeholderism [has] become a ‘rhetorical exercise aimed at neutralising criticism’ rather than a truly unique and participatory mechanism for governing a global resource” (ibid, p. 642).

Furthermore, she identifies three major stakeholders within this regime: *government*, *private sector*, and *civil society*. There seems to be a recurring *triadic model* within regulation and governance studies; Abbott and Snidal have named it the “governance triangle” (2009), which acts as a “heuristic device to structure analysis of widely varying forms of governance” (ibid, p. 52). According to the authors, this triangle consists of various zones, depending on the number of stakeholders involved in the deliberations, and each zone has a unique or mixed regulatory framework. Similarly, they also group actors in the same fashion as mentioned earlier: states, firms, and non-governmental organisations (NGOs).

However, we would be remiss not to highlight the consequences of discussing governance structures that refer only to these three actors: it normalises and reinforces a governance imaginary, where outsiders are excluded of the balance and, thus, risks replicating power imbalances and a quasi-elitist power structure. Additionally, the dynamics produced among these actors are contentious, which the authors often describe as “[a] transnational arena” or “bargaining game” ( Abbott & Snidal, 2009, p. 48), painting a picture of struggle for domination. Again, in a more Habermasian interpretation, the idealised exchange of rational arguments that, inevitably, will lead to a logical consensus ( Habermas, 1992), polycentric contention is framed as benign, constructing consensus, legitimacy ( Black, 2008) and fairness.

Yet, Carr argues that such an interpretation *neutralises* attempts to further politicise the discussion in regard to polycentricity, even if “[it celebrates] the inherently conflictual nature of ‘the political’” ( Cammaerts & Mansell, 2019, p. 5). The primary reason for this critique is that there are deep asymmetries in the relations that shape multi-stakeholderism and, as such, there cannot exist a fair exchange of ideas. Similarly, Cammaerts and Mansell call for a “radical turn” to pluralism, one that will have the “generative discursive power to render visible asymmetries and biases” of governance structures and, specifically, platform governance (ibid, p. 15). Following suit, this article argues that, on the one hand, we ought to further theorise about multi-stakeholderism instead of taking its dominance for granted and, on the other, that we have to expand our analytical and conceptual models of governance to account for these asymmetries and biases.

## Internet and platform governance

### Internet governance

The term “internet governance” dates back to the years after the commercial internet’s birth, circa mid 1990s; as Brousseau and Marzouki note, one of the earliest uses of internet governance, “as a tentative political construct” (2012, p. 2), was observed in the 1998 International Telecommunication conference. The reason why the authors label it as a political construct is because, up until that point, the term “internet governance,” was mostly related to technical issues of the internet, albeit a not well-known one. It was during that time that a specific socio-political agenda was also identified, along with its surrounding stakeholders ( Brousseau *et al.*, 2012, p. 4). Certainly, even within those fora, actors could not entirely agree on the exact nature of participating stakeholders. Brousseau and Marzouki paint a picture of a dichotomy between the “technical community,” who were defensive of the internet’s principles and values that would be ensured by self-regulating institutions and the “civil society,” that identified social actors and “commonly defined rules” outside the strict “Internet community” as crucial (ibid).

A few years later, in 2006, the United Nations (UN) founded the Internet Governance Forum (IGF). This marked a new era for internet researchers and, largely, the internet’s modus operandi ( Hofmann *et al.*, 2016, p. 3). The IGF provided us with the first formal definition of internet governance: “Internet governance is the development and application by Governments, the private sector and civil society, in their respective roles, of shared principles, norms, rules, decision-making procedures and programmes that shape the evolution and use of the Internet.” This multi-stakeholder framing has truly been the cornerstone of internet research ever since. It should be noted, though, that this paper does not confine the framing of internet governance to the infrastructural or computational aspect of the internet ( ten Oever, 2020, p. 27) but expands it to account for the broader stakes that are at play, like the internet’s “private ordering” ( DeNardis, 2010). As a result, we are mainly interested in exploring the “manifestations of power and political values” ( Hofmann *et al.*, 2016, p. 4) of participating actors colliding one with another, co-shaping governance.

### Platform governance

More recently, a discussion concerning a new chapter in the multi-stakeholder internet governance model has emerged, that of that of online content regulation ( Douek, 2021) within what some have named the “platform governance” ( Gorwa, 2019b; Helberger, 2020). A satisfactory definition of platform governance is given by Gorwa as “an approach necessitating an understanding of technical systems (platforms) and an appreciation for the inherently global arena within which these platform companies function” ( Gorwa, 2019b, p. 5). So, platform governance entails the study of governance of platforms (i.e., how platforms participate in multi-stakeholder governance structures and how regulation is developed by these structures and applied to platforms), as well as by platforms (i.e., how platforms themselves govern their spaces through self-regulating mechanisms like Community Guidelines).

However, this definition principally addresses the so-called “Big Tech” platform companies (also called GAFAM – Google, Amazon, Facebook, Apple, Microsoft), perpetuating thus a narrow understanding of the modern internet as a space occupied by a handful of oligopolistic firms, which leaves little space to “ reimagine the internet” and reinforces the idea that recent regulation is primarily shaped around “Big Tech” companies, which replicates existing power asymmetries ( Carr, 2015). Even though this may very well be true, we ought to be critical of the rhetorical vehicles we choose to build our conceptual work. As a result, we should approach platform governance as distinct from the broader internet governance structure or, perhaps, as a sub-field; it is important not to conflate the internet with private platforms, as that would narrow our perspective in a rather platform deterministic fashion ( Caplan *et al.*, 2020).

Nevertheless, platform governance holds significant value both on a conceptual and on a practical level: the former because as a means to think of the ways in which stakeholders pertaining to private platform companies participate in shaping governance and regulation, while the latter to gauge and study the various agreements or collaborations forged and shaped among stakeholders. In that sense, platform governance helps us to demarcate the field, as well as the object of inquiry and, thus, serves as a useful analytical lens to study governance structures that involve platforms.

 Furthermore, the present platform governance status quo seems to be shifting away from self-regulatory frameworks to complicated collaborative ones ( Gorwa, 2019b; Helberger, 2020). We reckon that the main reason behind this change, is the desire of private actors with strong “opinion power” ( Helberger, 2020) to ensure public legitimacy and fend off public intervention, thus, ensuring self-governance, that is the ability to function independently of public audit and accountability. Moreover, as Evelyn Douek sharply notes, “platforms […] [play] catch-up to societal demands for more responsible content moderation through self-regulatory innovations and reforms” (2021, p. 4; emphasis added).

For instance, when Facebook publishes guidelines on online content regulation, this should be seen as a move to formalise their content moderation processes by welcoming collaborations with other stakeholders and, thus, mitigating part of their responsibilities. However, a problem that quickly arises with this approach, and which Natali Helberger hints at with the “opinion power” concept (2020), is that it obscures or, at least, downplays the governance conflicts. Where internet governance was imagined to be a self-governed and self-regulated space, platform governance is imagined as a space of co-governance and co-regulation. Inviting co-governance and, consequently, co-regulation is, of course, not reproachable, quite the contrary; Douek believes that public regulation can make systems of content regulation “more accountable and credible” (2021, p. 59). Still, we should see such invitations as part of their communication strategies aimed at building legitimacy and affecting accountability ( Black, 2008, p. 151).

### An operational framework to study platform governance

**Figure 1.**
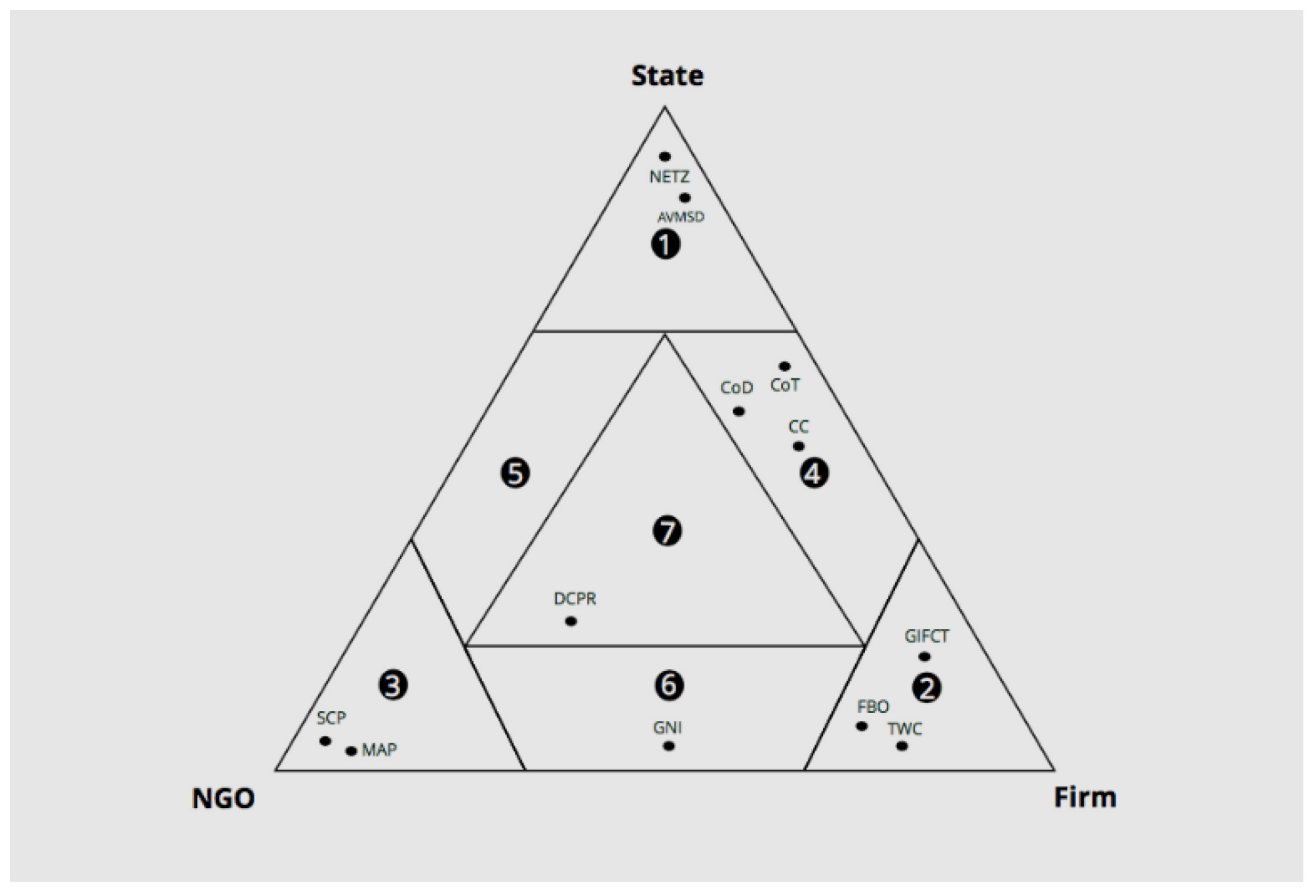
Robert Gorwa's formulation of the ‘Platform Governance Triangle’ depicting the EU content regulation landscape ( Gorwa, 2019a, p. 7).

Circling back to the conceptual merits of platform governance, Gorwa, following the political science tradition, has suggested to study platform stakeholders’ relations through their outcomes (i.e., regulations or agreements). To that end, he suggests exploring these products using the “platform governance triangle” ( Gorwa, 2019a), which is a re-contextualisation of Abbott and Snidal’s governance triangle, in which each corner represents a stakeholder: NGO, state, and firm ( Figure 1). Gorwa also offers a rather comprehensive view of different platform regulations that indicate governance relationships among stakeholders.

So, while the governance triangle serves as a valuable conceptual model of pinpointing stakeholders, it restrains us from having a more nuanced picture. However, it should be mentioned that Robert Gorwa’s re-framing of the triangle to illustrate the European content regulation landscape ( Gorwa, 2019a, p. 7), adds some nuance concerning the stakeholders’ relations concerning online platforms’ regulation. Nonetheless, we argue that there are some stakeholders that are difficult to group together and that clustering them solely based on a ‘spatial’ manner (i.e., where they stand in the governance triangle) does not do justice to their unique nature.

It is becoming increasingly difficult to distinguish actors’ interests in governance deliberations; for instance, private actors, ranging from news media organisations to platforms, have competing interests that are difficult to account for with the governance triangle model. Moreover, new stakeholders, like users or citizens, have been playing an important role in these deliberations. For instance, not only have they been participating in regulatory consultations (e.g., Commission’s Public Consultations) but they have also been involved in community-led platform governance structures; for example, Facebook Oversight Board has been receiving comments from the broader public to inform their decision-making process.

As a result, although the “governance triangle” lens is indeed useful in identifying the overarching actors at play (public, private, and NGOs), it would benefit from a reiteration. As governance regimes are becoming increasingly complex, so does the need for a more nuanced approach to identifying participating stakeholders. For instance, Flew *et al.*, criticise Gorwa’s triangle as not allowing us to conceive “inter-capitalist interests” ( Flew *et al.*, 2021, p. 129), following the recent Australian “ Mandatory News Media Bargaining Code,” that would require Facebook and Google “to collectively negotiate with commercial news publishers for payment for the use of the news content they carry” (ibid, p. 128).

This is why, this paper wishes to conclude this literature review by proposing an expansion of that triangle to account for “the shifting allegiances across categories and also the heterogeneity of interests within them” ( Flew *et al.*, 2021, p. 134). In particular, it suggests to start studying clusters of stakeholders through their relations (procedural approach to governance) and not only based on their institutional profile (normative/outcomes-oriented approach).

**Figure 2.**
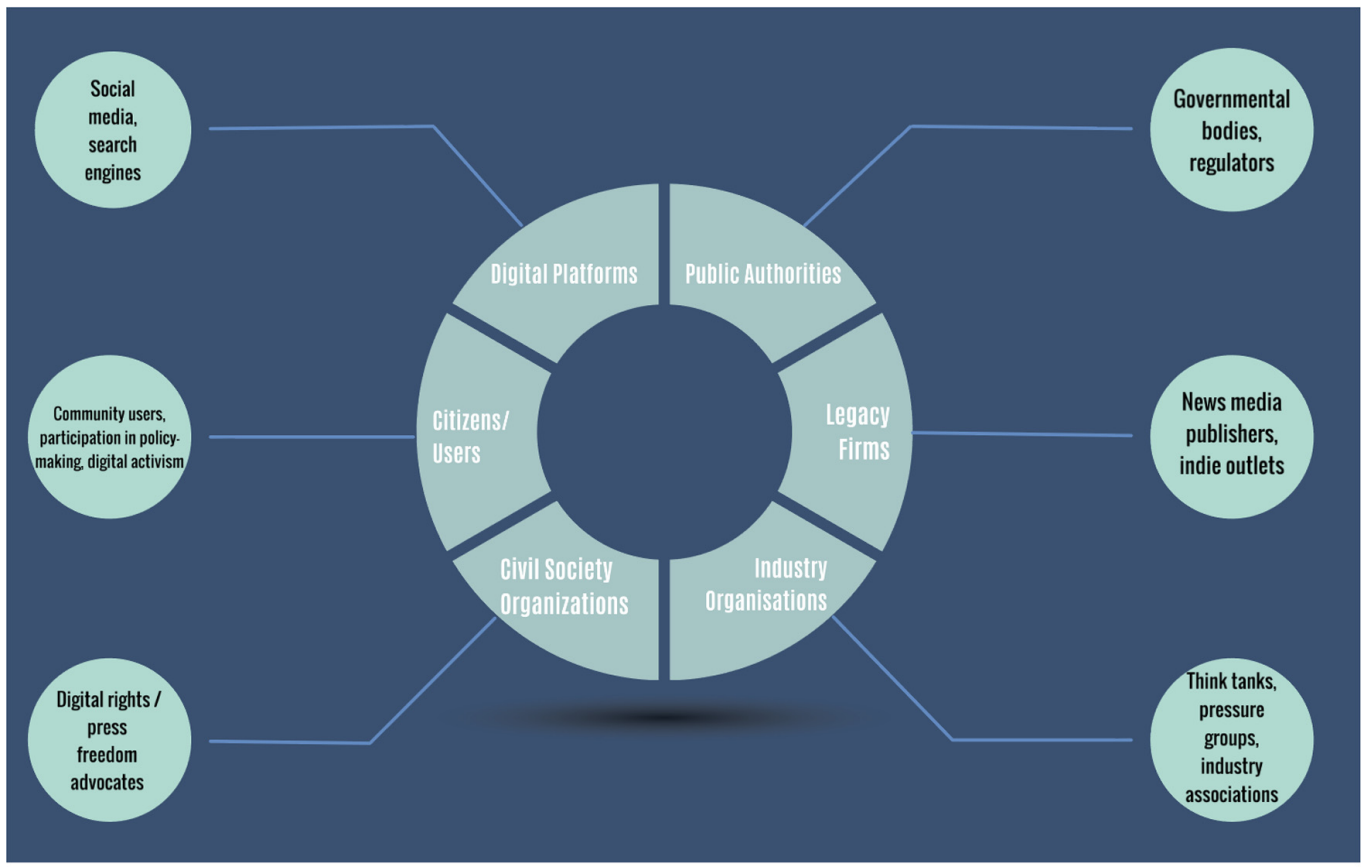
Expansion of platform governance triangle.

As a result, we propose that a more suitable concept would be that of “governance clusters,” ( Figure 2) which are comprised of actors sharing some common fundamental principles and interests. The governance clusters presented in the formulation below is merely an operationalisation of the governance triangle to account for subtle nuances that exist within modern governance structures. Therefore, we do not propose to abolish the traditional model of triangle but rather elasticise it, so as to accommodate more complicated arrangements. Also, this figure illustrates a platform governance structure within the context of the news industry; so, other instances of platform governance are not accounted for here but we believe that could be explored using the operationalisation proposed here. It should also be noted that the clusters’ size does not represent their importance or power; asymmetries and biases exist in this illustration as well.

Moreover, it takes into consideration the bifold quality of governance as structure and governance as power that explained earlier: that is, it allows us to map relevant stakeholders that were not visible in the triadic model, as well as to analyse the power relations of stakeholders – and their asymmetries. Consequently, it considers both the organisational or regulatory arrangements among stakeholders and the procedures that underpin these arrangements. Following the critique of Flew *et al.*, we’d like now to suggest how the operationalisation of the platform governance triangle proposed here could be used to study governance deliberations around Australia’s new regulatory framework, News Media Bargaining Code: (i) public authorities, (ii) digital platforms, (iii) civil society organisations, (iv) legacy firms, (v) industry organisations and, last, (vi) citizens/users. We’d like to briefly expand on each stakeholder to avoid misconceptions:

i. **Digital Platforms**: there is a whole scholarly debate on the elusive definition of platforms. A satisfactory definition is that: “An online ‘platform’ is a programmable digital architecture designed to organise interactions between users - not just end users but also corporate entities and public bodies” ( van Dijck *et al.*, 2018). This paper is first and foremost interested in social infomediaries ( Smyrnaios & Rebillard, 2019) and social media platforms that host, curate and disseminate content online ( Gillespie, 2018) and not other types of platforms, like data brokers or advertisers.
ii. **Public authorities**: refer to public actors, like governmental, national or supranational organisations, who are either elected or appointed by elected officials, with the authority to pass regulations, policies or legislation.
iii. **Civil Society Organisations**: Tjahja *et al.* (2021) have provided us with an illuminating discussion and typology of what exactly civil society means in a modern multi-stakeholder governance network, namely that of the Internet Governance Forum, highlighting the contested nature of the concept. One of the most important elements is their framing of civil society organisations as “intermediaries” advocating for their “communities’ interests” ( Tjahja *et al.*, 2021, p. 3). Subsequently, in this operationalisation, civil society mainly refers to organisations that engage in advocating for citizens and users’ digital rights, trying to hold to account both public authorities and social media.
iv. **Legacy firms**: refers primarily to news organisations that play an active role in shaping the regulatory agenda of online content. Platforms might argue that news’ revenue is “ minimal,” but their role in platform governance is crucial ( Napoli, 2015; Smyrnaios & Rebillard, 2019) because, among others, they make platforms nodes of public interest, where information is centralised ( Helmond, 2015). Additionally, ever since the consolidation of online platforms, news organisations have been trying to stay afloat and retain or increase their visibility. To that end, many news organisations have struck different deals with online platforms, while others have been pushing their associations to either collectively negotiate with platforms or push public authorities to intervene.
v. **Citizens/users**: these are theoretically represented in the governance deliberations by civil society organisations ( Regilme, 2018). yet, we believe that this could be more of a hypothesis rather than an axiom. In other words, representation in modern networked governance could be questionable and, thus, public consensus could be nothing more than wishful thinking ( Flyvbjerg, 1998, pp. 214, 229), What is more, in modern deliberations, we see citizens participating individually and directly: for instance, the European Commission has put public, open consultations in place, where every stakeholder can participate to help officials draft regulations. Finally, there exist platforms, like Wikipedia or Reddit, that rely on their users to moderate their content. Finally, social media influencers could also be a form of opinion makers, as people making a living off monetising their popularity on social media, which is integral to platforms’ economy; yet, their role as governance stakeholders, along with the way that platforms treat them, has been understudied ( Caplan & Gillespie, 2020) and underestimated by other stakeholders. So, in such complex governance arrangements, we ought to take into account citizens, as well as users distinctly.
vi. **Industry Organisations**: we primarily refer to industry associations, think tanks or lobby groups, that work to their respective industry’s interests. We propose to look at them separately from civil society organisations because they do not work for the public interest but, rather, their private interest. Finally, we call them opinion shapers because they can communicate their industry’s interests to all other stakeholders, either independently or not, and can, thus, shape the governance stakeholders’ opinion on issues of regulatory deliberation.

So, when the Australian government following the report of the Australian Competition and Consumer Commission (ACCC), passed a bill that would force leading platforms Google and Facebook to negotiate with news publishers for news content hosted by their services, it was seen as an intervention to the platform governance’s status quo. This is why the platforms’ reactions, especially from Facebook, was so strong, which resulted in blocking even governmental agencies that were informing the public regarding the development of the coronavirus pandemic and, thus, involving citizens to the governance deliberations, even if indirectly. This could be why the Australian government agreed to some amendments demanded by platforms.

Moreover, civil society groups and opinion shapers participated in the consultations held by the ACCC ( Flew *et al.*, 2021), albeit the former in a much limited manner (ibid, p. 128). It worth merits that the stakeholder analysis proposed by Flew & Lim (2019, pp. 541–574) suits great the operationalisation proposed here to study governance deliberations and power relations. Last, news media, apart from being directly involved in the deliberations, used their own means to sway public discourse, while large legacy media organisations were negotiating other financial deals directly with platforms. Finally, as mentioned earlier, the proposed operationalisation allows to account for inter-stakeholder competing interests: for instance, smaller Australian news media organisations voiced their concerns over the Code’s advantageous position that it gives to large corporate publishers at the expense of smaller players. Concluding, this short example sought to emphasise how increasingly complicated governance structures are becoming, as well as to illustrate how, by expanding our understanding of stakeholders, we can better study governance procedures.

## Conclusion

This paper sought out to introduce a comprehensive overview of literature concerning the concepts of regulation and governance, as well as to connect them to the scholarship engaging with the study of platform regulation and platform governance. Specifically, the paper introduced the various approaches to defining regulation and governance in tandem with multi-stakeholderism, and then proceeded with connecting said notions, primarily, with two research fields: internet governance and platform governance. This was chosen so as to emphasise the importance of differentiating between the two fields in an attempt to avoid conflating the internet with digital platforms. First, we defined regulation as a governance mechanism, involving the intentional – direct or indirect - intervention in the activities of a stakeholder, with the intention to change that stakeholder’s modus operandi, which, in turn, may have unpredictable – yet measurable - consequences to the governance regime Then, we defined governance as a complex networked structure that accommodates different stakeholders that are connected and coordinated through various types of regulations, norms and practices; what is more, we also explained how governance holds a bifold analytical quality that allows for the exploration of a governance regime’s structure and the power relations that underpin it.

Furthermore, we made a brief historical overview of how the concept of internet governance was developed inherently tied to the notion of multi-stakeholderism and how it evolved over time to an important analytical lens to study the internet’s political stakes. The key takeaway point is that multi-stakeholderism has monopolised scholars and policymakers so much so that any critique towards the model is perceived as an attack to democracy or plurality ( Carr, 2015), which has led to the weakening of critical analyses and has perpetuated power asymmetries.

Last, we discussed the field of platform governance, which allows us to study the governance *of* platforms and *by* platforms within multi-stakeholder governance structures; we acknowledged the analytical value of platform governance as a way of studying ubiquitous digital platforms but also highlighted the need to avoid conflating them with the internet, especially the very large online platforms that have come to dominate the modern public sphere. As a result, we argued that the platform governance, both as a field of study and as a governance structure, poses an existential risk to the internet governance, precisely because of the domination of private platforms both as actors and as actants (i.e., field of scholar study and centre of policy-making).

Moreover, we proposed to adjust the platform governance triangle, that was proposed by Gorwa (2019b) and built upon the work of Abbott & Snidal (2009), in order to account for the increasingly complicated platform governance structures. We suggested an operational framework designed to study the recent Australia’s New Media Bargaining Code focusing on invisible clusters of governance stakeholders. We feel that this adjustment is necessary to: a) consider actors that do not fall in traditional categories (i.e., state, firm, NGO) due to their hybridity (e.g., Wikimedia is such an example), b) consider inter-stakeholder competing interests, and c) consider the importance of citizens and users in modern networked structures.

Future research should look into studying the new governance clusters in concert to developments in the online content regulation front. For instance, citizens’ contributions to governance deliberations through the European Commission’s public consultations or academics’ participation in panels aimed at designing or assessing regulatory frameworks. In addition, much more detailed work is needed to theoretically underpin the emerging field of platform governance, while a systematic literature review could certainly help us understand when and how the term gained traction. As hinted throughout the paper, platform governance enables us to explore the implications of online content regulation and the governance deliberations to the public discourse and the public sphere in general ( Papacharissi, 2002; Salikov, 2018). Yet, we ought to be aware of its limitations and risks. We believe that there is a timely need to adjust the conceptual tools we have in such a way that avoids perpetuating power imbalances and accounts for the complex multi-stakeholder governance structures; this is crucial to work toward a much-needed reimagination of governance that reinforces polycentricity and decentralisation.

## Data availability

All data underlying the results are available as part of the article and no additional source data are required.
