# Supplementary material for: The existential stakes of platform governance: a critical literature review
Source: Open Res Eur. 2021 Jul 1;1:31. Originally published 2021 Mar 31. [Version 2] doi: 10.12688/openreseurope.13358.2 (PMC10445813; doi:10.12688/openreseurope.13358.2)
Supplement: Peer review history

# Peer review: Reviewer response for version 2

**DOI:** 10.21956/openreseurope.14946.r27201

**Published:** 2021-07-09

**Authors:** João Carlos Magalhães

**Affiliations:** 1 Alexander von Humboldt Institute for Internet and Society, Berlin, Germany; 2 University of Groningen, Groningen, The Netherlands

I commend the author's revisions, which essentially answer my initial comments.

 Four elements that the author might consider in future iterations of this text:

1. The differentiation between regulation and governance. While the current version is much clearer than the first, I find it hard not to see both concepts as different ways of talking about power. What raises the question of why, after all, we should use  terms like "regulation" and "governance" when we are trying to understand "power". This is hardly an issue that is particular to this manuscript, though.
2. How the conceptualisation of regulation and governance informs the definition of platform governance. It would be important to be a bit more explicit, and to be sure that the critical discussion of those two concepts arrive at conclusions that are clearly used / discarded when defining platform governance. In this way, the reader will certainly find the flux of ideas much easier to grasp.
3. Materiality. Platforms are new in various ways but, mostly, because they are materially organised around datafication. This is not made as explicit as it could -- and arguably should -- be. Bringing datafication to the table can make the theorisation work more challenging, but also more rewarding.
4. Lastly, I encourage the author to keep refining these ideas with a view to not only "review" the literature but also produce his own concepts -- something that is already partially done here, clearly.

If any, are all the source data and materials underlying the results available?

No source data required

Is the work original in terms of material and argument?

Partly

Is the work clearly and cogently presented?

No

Is the argument persuasive and supported by evidence?

No

Does the research article contribute to the cultural, historical, social understanding of the field?

Partly

Does it sufficiently engage with relevant methodologies and secondary literature on the topic?

Partly

Reviewer Expertise:

Media, power, ethics, datafication, democracy, platforms

I confirm that I have read this submission and believe that I have an appropriate level of expertise to confirm that it is of an acceptable scientific standard.

# Peer review: Reviewer response for version 2

**DOI:** 10.21956/openreseurope.14946.r27207

**Published:** 2021-07-07

**Authors:** Terry Flew

**Affiliations:** 1 Department of Media and Communication, The University of Sydney, Sydney, Australia

This paper makes two very important contributions to current debates about Internet regulation and platform governance. The first is that it helps to clarify the important conceptual distinction between regulation and governance. There is a tendency in the literature to assume that we have moved from regulation to governance based on complexity, actor-network theory etc. But what is apparent is that the regulation literature can flexibly incorporate a wide range of practices, many of which are considered to be governance e.g. ‘nudging’. The point that ‘these two approaches are not oppositional one to another but rather highlight different aspects of governance’ is well made. 

 One point that could have been picked up is the inevitability of governance for platforms. By the nature of their operations in multi-sided markets, they have to be engaged in governance, and also to work with multiple stakeholders simultaneously. In that way, governance is part of the business model of platform businesses. It is what is new and distinctive that needs to be discussed, much of which sits within the sphere of regulation. 

 I was broadly sympathetic to the critiques of multi-stakeholder governance, but felt that they need to be differently expressed. There are two different critiques being developed here: one that it is too much grounded in a ‘Habermasian’ rationalism, and the other is that it disguises the extent to which power relations really construct global Internet governance. If one goes back to early debates about multi-stakeholder governance, it is arguable that 'Habermasian' ideas had much influence at all. In many respects, the key motivator was to keep nation state governments at arm’s length. In that respect, enlisting NGOs has proven to be of ongoing utility, whether or not they actually have power in the framework. 

 The other key contribution was to argue that we need to move beyond a ‘triadic’ model for platform governance. The ways in which other players were included was particularly important e.g. bringing in other media companies as agents in their own right, and regulators as potentially having agency in shaping the debates. As the earlier review did, I wondered about the citizens/users category. Are these in effect de facto civil society organisations? The literature on political agency indicates that a political agent needs to be represented, and have a capacity to turn such representation into action e.g. ‘the working class’ is not a political agent, but trade unions that purport to represent them are. 

 I would also agree that governance needs to be extended beyond the content area. There is no discussion here of competition policy, for instance, but it is clearly at the forefront of contemporary debates about Internet regulation, as seen with the antitrust discussion in the US. There are also growing debates around data governance and AI, which include platform companies, but go beyond platforms.

If any, are all the source data and materials underlying the results available?

No source data required

Is the work original in terms of material and argument?

Yes

Is the work clearly and cogently presented?

Yes

Is the argument persuasive and supported by evidence?

Yes

Does the research article contribute to the cultural, historical, social understanding of the field?

Yes

Does it sufficiently engage with relevant methodologies and secondary literature on the topic?

Yes

Reviewer Expertise:

Digital platforms; platform governance; Internet regulation; media policy

I confirm that I have read this submission and believe that I have an appropriate level of expertise to confirm that it is of an acceptable scientific standard.

# Response

**Published:** 2021-07-09

**Authors:** Charis Papaevangelou

**Affiliations:** University Paul Sabatier - Toulouse III, France

Dear Mr. Flew, Thank you very much for your positive and detailed report. I really appreciate that you took the time to review my paper, which was heavily influenced by your own work. Thanks to the format of Open Research Europe, I would like to take this opportunity to briefly respond to three points you raised:

- Concerning the multi-sidedness of platforms, I totally agree and this is in fact an oversight of my part. I was too focused on the literature/theoretical synthesis of regulation & governance that did not actually expand a lot on the distinctiveness of platforms. As this is part of my PhD project, I will make sure to elaborate further on that. The point that governance is indeed part of platforms' business model is very crucial; commercial internet could not have been realised without the de-regulation period that established governance & multi-stakeholderism. In that sense, regulation could be seen as a way of re-affirming state power.
- Concerning your other point on civil society but also citizens/users, what I tried to say with my paper is that: although NGOs were included in multi-stakeholder governance structures they did not always serve the public interest (representing citizens). This is why I tried to make the distinction between civil society organisations, that often are characterised advocates or activists nowadays) and other NGOs, like industry associations. What is more, I also tried to emphasise the distinct role that citizens/users play in modern governance as stakeholders who may have direct (or, at least, indirect) impact on deliberations (e.g. through participating in public consultations or through participating in community-led platforms or, even, through flagging content on platforms).
- Last, your point on studying governance beyond content regulation/governance is indeed valid and one that needs to be further analysed. I did not wish to cover data or competition governance in this paper, but it is very timely do so, given the upcoming regulations (e.g. the Data Governance Act of the Commission). I also think that we could expand this to cover AI as well. It is just that I tried to offer a broader lens to study governance that could be then operationalised to study specific fields like the aforementioned.

I hope this clarifies some questions.  Again, thank you very much for your helpful feedback. Charis

# Peer review: Reviewer response for version 1

**DOI:** 10.21956/openreseurope.14428.r26723

**Published:** 2021-05-12

**Authors:** João Carlos Magalhães

**Affiliations:** 1 Alexander von Humboldt Institute for Internet and Society, Berlin, Germany; 2 University of Groningen, Groningen, The Netherlands

This manuscript discusses and tries to put into dialogue three closely connected concepts – regulation, governance and platform governance. It defines itself as a non-exhaustive “critical exploration of relevant literature” and “an invitation to investigate the interdisciplinarity and depths of” of the scholarship about the governance and regulation of online platforms, “as well as their implications relating to democracy and public discourse” (p. 1).

 Overall, this is a very welcomed effort as the field of platform governance has not been sufficiently theorised. However, the manuscript must undergo major revisions before a decision on its academic merit (and, thus, indexing) can be made.

 The perhaps primary problem is that the MS does not deliver what it promises. The MS’s plan appears straightforward: to discuss and define “regulation”, “governance” and their linkages, and then “connect” these two ideas to debates on platform governance (p. 9). However, it does not really offer a convincing conceptualisation of neither regulation nor governance. Regulation, it is said on page 5, is "a governance mechanism, involving the intentional – direct or indirect - intervention in the activities of a stakeholder, with the intention to change that stakeholder’s modus operandi, which consequently has unpredictable consequences to the rest of the governance environment” (p. 9). So, the definition of regulation hinges on that of governance. The problem is that MS’s view on governance remains elusive. On page 4, it says that it understands governance as “that politically charged notion that signifies ‘to govern’”, a literalistic interpretation that does not amount to a scholarly definition. Then, without reviewing other definitions, it appears to side with the Foucauldian definition of “governamentality”. This is an issue in itself since governamentality is a much broader theory, that regards the complex interactions between power and freedom (something the MS does not note). In fact, I could not find a moment in which the actual differences between "regulation" and "governance" are specified. On page 6, it is said that "we can imagine these multi-stakeholder environments ^1^ as contentious fora, where power relations among actors surface the interdependence of one another, while shaping the governing status quo. This consolidates the difference between regulation and governance, as well as their strong connection" - but I fail to see how this clarifies anything. What "consolidates" the difference? Is "consolidate" the right verb here? Where is "regulation" and "governance" in these "environments"? I sense that the MS wants to argue that "governance" means power, broadly understood, and "regulation" would be a much narrower form of power -- but this is far from clear. Since “regulation” and “governance” are not properly defined, the MS offers no clear articulation between these concepts and platform governance. There are hints that current studies of platform governance are not critical enough and may suffer from similar simplifications to debates on multistakeholderism, topics that are debated in the MS. Yet, one would expect a much clearer demonstration of how the MS’s discussion of those two concepts can inform and illuminate the definition of platform governance offered in the penultimate section – to which I turn now.

 Leaving aside the said lack of clear connection between the three concepts, the MS falls short of a proper conceptualization of platform governance – which is the MS’s other major issue. It is unclear why the MS assumes that platform governance can be restricted to “online content regulation” since platform governance involves many more areas (e.g. monopolistic practices, privacy/surveillance etc). Then, it proposes that platform governance should go beyond the “triangle” of states, private firms and NGOs, and lists six “clusters” of stakeholders. Yet, one could easily argue that, if by NGO one means *any* non-governmental organisation, five of those six “clusters” can be easily framed with the same “triangle”: digital platforms are private firms, “public authorities” are the state, and NGOs, news media, and “opinion makers” such as academics are representatives of non-governmental organisations. “Citizens” might indeed be a new category but we must remember that in most models citizens are said to be represented by both states and NGOs. Furthermore, even if we assume that there is analytical value in breaking the triangle into “clusters” (something that I am sympathetic to but the MS does not demonstrate), one could argue that MS actually does not go *far enough* in its specification exercise. There are several “clusters” missing: other platforms (think of the relationship between Facebook and Apple Store), advertising companies (which are platform’s real clients and hold enormous power over them), traditional Internet governance institutions (ICANN etc), internal divisions within platforms. It would be therefore crucial to present a justifiable criterion behind the zooming in movement – what is not done by the MS.

 There are other, more specific issues with the MS, and I have provided a minutely commented file. However, these two problems make it hard for me to understand what is the merit of this MS, and should in my view be prioritised.

 The author is of course free to take any revision route he finds useful to address the issues described above. My suggestion is that he uses the MS’s conclusion (which is much clearer than the rest of the text) as an initial blueprint, and then re-writes this piece so as to answer the following questions:

1. What is the MS’s definition of regulation, and how does it relate to other definitions of regulation?
2. What is the MS’s definition of governance, and how does it relate to other definitions of governance?
3. What is the relationship between the MS's definitions of governance and regulation? In which ways do they differ?
4. What is the MS’s definition of platform governance, and how does it relate to both the MS’s definitions of regulation and governance *and *existing definitions of platform governance?

I strongly advise the author to focus on the clarity of his argument, and justify his choices carefully.

 Finally, I would like to stress that, despite my criticism of the current state of the MS, the problem it tackles is quite important. The MS holds the potential of making an original contribution to a topic that is urgent and sparsely theorised.

If any, are all the source data and materials underlying the results available?

No source data required

Is the work original in terms of material and argument?

Partly

Is the work clearly and cogently presented?

No

Is the argument persuasive and supported by evidence?

No

Does the research article contribute to the cultural, historical, social understanding of the field?

Partly

Does it sufficiently engage with relevant methodologies and secondary literature on the topic?

Partly

Reviewer Expertise:

Media, power, ethics, datafication, democracy, platform.

I confirm that I have read this submission and believe that I have an appropriate level of expertise to state that I do not consider it to be of an acceptable scientific standard, for reasons outlined above.

# Peer review: Reviewer response for version 1

**DOI:** 10.21956/openreseurope.14428.r26684

**Published:** 2021-04-19

**Authors:** Philip Napoli

**Affiliations:** 1 Sanford School of Public Policy, Duke University, Durham, NC, USA

This article provides a concise and well-organized literature review related to the concepts of regulation and governance that inform and guide ongoing deliberations around the notion of platform governance and, in particular, the realm of online content regulation that has become an increasingly prominent component of contemporary platform governance deliberations.

 One comment I would make in regards to this piece's focus on online content regulation within the platform governance context is just to emphasize that platform governance is a more expansive concept that extends beyond the realm of content regulation. Indeed, what has been so striking over the past five years or so is to observe how the focus of platform governance debates and deliberations has shifted so dramatically, from what once was an almost exclusive focus on issues related to platforms' handling of user data to the much more prominent focus on content regulation that we see today. 

 This piece's efforts to theoretically unpack the concepts of regulation and governance are a valuable exercise that will benefit scholars who are new to the platform governance field; it also serves as a useful reference point for scholars who have been working in this area for some time.

 And, of course, while there are practical limits to how much literature can be synthesized in an effort such as this to provide a concise overview and critical analysis, I think readers of this piece might find Manuel Puppis' work on media governance ^1^ a useful resource to consider as well, given that it is, to some degree, a precursor to the type of analysis presented here (though with a different contextual focus).

 I think the points that the author makes about the need to unpack the notions of regulation and governance, and the need to study the space between regulation and governance, are particularly important, as there does appear to be the occasional tendency amongst scholars to use the two terms without the necessary precision.

 Along similar lines I appreciate the effort to contextualize platform governance within the more established realm of Internet governance. However, I don't know if I would want to see platform governance conceptualized, as the author suggests, as "not so much as a subfield of Internet governance, but rather its evolution." This is a statement that, for me, evokes the widely criticized tendency by Facebook's Mark Zuckerberg, to argue that we need new rules for the Internet - with the critique arising from the fact that many of the problems requiring attention have emerged specifically from the digital platforms such as Facebook that make use of the Internet, but that do not comprise the Internet. This strikes me as an important distinction to maintain, especially when we talk about content regulation, given that content regulation debates tend to ignore the fact that content removed from, or restricted on, social media platforms can still exist elsewhere online. So, my point here is that I am nervous about any analytical approach that appears to move us in the direction of conflating the Internet with digital platforms, regardless of how much these digital platforms have come to dominate the Internet at this point.

 The author's efforts to lay out the key stakeholder groups involved in platform governance are particularly useful. However, I would like to explore a bit the "citizen" category as described in the text and reflected in Figure 1. Specifically, I'm interested in whether there is a meaningful distinction that needs to be made here between citizens and users. As laid out in the text, citizens participate in the governance process through being represented by civil society organizations and through direct participation in regulatory proceedings. What seems to be left out in this framework are the ways in which digital platforms occasionally rely upon their user base to assist in the governance process, in ways such as relying upon users to flag problematic content, or drawing upon survey or user behavior data to inform or justify shifts in organizational policy, algorithmic design, etc. Within these contexts we are talking about a narrower subset of the populace than citizens (which itself raises interesting implications about the distribution of platform governance influence within the population).

 Similarly, I think that there is an additional dimension to the role of the news media as a stakeholder in the dynamics of platform governance that merits consideration. Specifically, I think it is important to acknowledge the increasingly influential role that the news media play as, essentially, platforms' fourth estate - that is, as key watchdogs of the digital platforms. It is investigative reporting and data journalism that have made public many instances of platform misbehavior, and abuses of the platforms by third parties. This has led some observers to describe the news media as a key content moderator for digital platforms. And there is certainly evidence that platforms have changed their behaviors in response to many of these reports. So I think this aspect of the increasingly complex relationship between digital platforms and the news media merits mention within the context of discussing the news media as a stakeholder in the process of platform governance, particularly given the extent to which platforms are simultaneously becoming an increasingly important source of financial support for news organizations in many national contexts.

If any, are all the source data and materials underlying the results available?

No source data required

Is the work original in terms of material and argument?

Yes

Is the work clearly and cogently presented?

Yes

Is the argument persuasive and supported by evidence?

Yes

Does the research article contribute to the cultural, historical, social understanding of the field?

Yes

Does it sufficiently engage with relevant methodologies and secondary literature on the topic?

Yes

Reviewer Expertise:

Media regulation/policy; platform governance; local journalism, media audiences.

I confirm that I have read this submission and believe that I have an appropriate level of expertise to confirm that it is of an acceptable scientific standard.

## References

[1] : Media Governance: A New Concept for the Analysis of Media Policy and Regulation. *Communication, Culture & Critique* .2010;3(2) : 10.1111/j.1753-9137.2010.01063.x 134-149 10.1111/j.1753-9137.2010.01063.x

# Response

**Published:** 2021-06-23

**Authors:** Charis Papaevangelou

**Affiliations:** University Paul Sabatier - Toulouse III, France

Dear Mr. Napoli,

 First, I would like to thank you for taking the time to review my paper and for approving it for publication. After a few months of revising the paper's initial version, I have to say that I think that it's now become a stronger piece, and I'd like to thank you for that. Your suggestion to look at Manuel Puppis' work was enlightening, as his work on media governance allowed me to better articulate what I was trying to say in my first version: governance can be a very strong analytical concept to study media (and platform) regulation! I think that governance has been predominantly used as a way of describing a set of relations from a more structural standpoint, losing thus part of its analytical capacity. What is more, your comment on the significance of not treating the internet as a space that consists of digital platforms, really resonated with me and what I was also trying - unsuccessfully - to argue in the first place. I also found your comments on the news media organisations as a governance stakeholder really encouraging to continue pursuing that direction of analysis, especially as the discussion about journalism's sustainability is becoming prominent around the world. All in all, your critique was very constructive and allowed me to make this paper better. It is important to note that this work is part of a larger PhD project studying the political economy of digital platforms' regulation; as such, it would be fair to say that your feedback made my thesis's theoretical framework better.
